# Identification and Characterization of *oriT* and Two Mobilization Genes Required for Conjugative Transfer of *Salmonella* Genomic Island 1

**DOI:** 10.3389/fmicb.2019.00457

**Published:** 2019-03-06

**Authors:** János Kiss, Mónika Szabó, Anna Hegyi, Gregory Douard, Karine Praud, István Nagy, Ferenc Olasz, Axel Cloeckaert, Benoît Doublet

**Affiliations:** ^1^National Agricultural Research and Innovation Centre, Agricultural Biotechnology Institute, Gödöllõ, Hungary; ^2^ISP, Institut National de la Recherche Agronomique, Université de Tours, UMR 1282, Nouzilly, France

**Keywords:** *salmonella* genomic island 1, integrative mobilizable element, IncA/C plasmids, origin of transfer (*oriT*), horizontal gene transfer, mobile genetic element (MGE), antibiotic resistance (AR)

## Abstract

The integrative mobilizable elements of SGI1-family considerably contribute to the spread of resistance to critically important antibiotics among enteric bacteria. Even though many aspects of SGI1 mobilization by IncA and IncC plasmids have been explored, the basic transfer elements such as *oriT* and self-encoded mobilization proteins remain undiscovered. Here we describe the mobilization region of SGI1 that is well conserved throughout the family and carries the *oriT*_SGI1_ and two genes, *mpsA* and *mpsB* (originally annotated as S020 and S019, respectively) that are essential for the conjugative transfer of SGI1. *OriT*_SGI1_, which is located in the vicinity of the two mobilization genes proved to be a 125-bp GC-rich sequence with several important inverted repeat motifs. The mobilization proteins MpsA and MpsB are expressed from a bicistronic mRNA, although MpsB can be produced from its own mRNA as well. The protein structure predictions imply that MpsA belongs to the lambda tyrosine recombinase family, while MpsB resembles the N-terminal core DNA binding domains of these enzymes. The results suggest that MpsA may act as an atypical relaxase, which needs MpsB for SGI1 transfer. Although the helper plasmid-encoded relaxase proved not to be essential for SGI1 transfer, it appeared to be important to achieve the high transfer rate of the island observed with the IncA/IncC-SGI1 system.

## Introduction

Conjugation is a widespread mechanism of horizontal gene transfer among naturally occurring plasmids and genomic islands. During conjugation, DNA is transferred from donor to recipient through a close cell-to-cell contact. Based on Gram-negative plasmid models, the process starts with the assembly of a multi-protein complex called the relaxosome around the origin of transfer (*oriT*). *OriT* is a *cis*-acting DNA region that is required for initiation of the transfer. The key enzyme of transfer initiation is the relaxase, which cuts either strand of *oriT* DNA at the *nic* site. The relaxase remains covalently bound to the 5′-end of the cleaved strand, which is subsequently delivered to the recipient across the membrane-associated DNA transport machinery, the type IV secretion system (T4SS). Initiation of conjugation often requires several auxiliary proteins implicated in the relaxosome (De La Cruz et al., 2010; Bellanger et al., 2014). The relaxosome is then recruited to the T4SS with the assistance of the membrane-associated coupling proteins (T4CP), which binds the cognate T4SS and the relaxosome complex through the relaxase or auxiliary proteins (Llosa and Alkorta, 2017). Based on phylogenetic analyses of relaxases, conjugative systems have been classified into eight major MOB families, however, numerous unclassified systems have also been reported (Garcillán-Barcia et al., 2009; Bellanger et al., 2014; Li et al., 2018). Some of these families include plasmids and mobile genomic islands (MGIs) as well (e.g., MOB_H_, MOB_C_, MOB_P_, MOB_V_, MOB_T_), indicating the relationship of the conjugative systems of the two groups of mobile elements.

In addition to resistance plasmids, MGIs are the major players in dissemination of multidrug resistance (MDR) phenotype among bacteria. MGIs provide selective advantages to their host by carrying resistance-, pathogenicity-, metabolic pathway- or symbiosis-related genes. After acquisition, MGIs integrate into the host chromosome by site-specific-, transposition- or homologous recombination to ensure their maintenance and vertical transmission (Bellanger et al., 2014). MGIs are classified into two major groups: integrative conjugative elements (ICEs, some of them were formerly known as conjugative transposons, (Liu et al., 2019) and integrative mobilizable elements (IMEs). In addition to the chromosomal excision/integration ability, ICEs encode for their own conjugation system and are fully autonomous in horizontal transfer (Carraro and Burrus, 2014), while IMEs have a limited set of transfer genes and thus require the presence of other conjugative helper elements to hijack their transfer systems (Douard et al., 2010, Daccord et al., 2013; Bellanger et al., 2014).

Many studies about MGI mobilization unravel their integration and excision reactions. However, the mechanism of their conjugation is less investigated, and their transfer genes are mostly identified by their similarities to those of conjugative plasmids. Like all conjugative and mobilizable plasmids, MGIs also carry their own *oriT* sequence (Bellanger et al., 2014). Most *oriT* sequences have been identified in conjugative plasmids, where they are often located adjacent to genes of the relaxosome components (e.g., in RP4, R388, R6K, pCW3, pIP501). Although conserved sequence motifs have been found in *oriT*s close to the *nic* site of IncPα, Ti, Ri, R64 and pTF-FC2 plasmids (Pansegrau and Lanka, 1991), *oriT*s are generally diverse sequences [*oriT*DB (Li et al., 2018)], which frequently contain inverted repeat (IR) motifs and AT-rich regions [F (Frost et al., 1994), R6K (Avila et al., 1996), IncPα (Fürste et al., 1989),(Pansegrau et al., 1994), pAD1 (Francia and Clewell, 2002), pAM373 (Francia and Clewell, 2002), pCW3 (Wisniewski et al., 2016) and IncA and IncC plasmids (Hegyi et al., 2017)]. Transfer systems of several ICEs have also been analyzed in details and the *oriT* has been identified in Tn*916* (Jaworski and Clewell, 1995), Tn*4445* (Smith and Parker, 1998), ICE*Bs1* (Lee and Grossman, 2007), SXT/R391 (Ceccarelli et al., 2008), ICE*clc* (Miyazaki and Van Der Meer, 2011), ICE*hptfs4* (formerly referred as tfs4) (Grove et al., 2013) and the related IMEs mobilized by SXT (Daccord et al., 2010, 2013; Li et al., 2018). Although all *oriT*s contain several IR motifs, no extensive sequence similarities can be observed between *oriT*s of unrelated elements. In contrast, IMEs hijacking the same helper elements have similar *oriT*s to each other and to that of the helper (Daccord et al., 2010). Most conjugative plasmids and MGIs have a single *oriT*, however, in some cases two separate and functional *oriT*s have been identified [pAD1 (Francia et al., 2001; Francia and Clewell, 2002), R6K (Avila et al., 1996), ICE*clc* (Miyazaki and Van Der Meer, 2011)].

**FIGURE 1:**
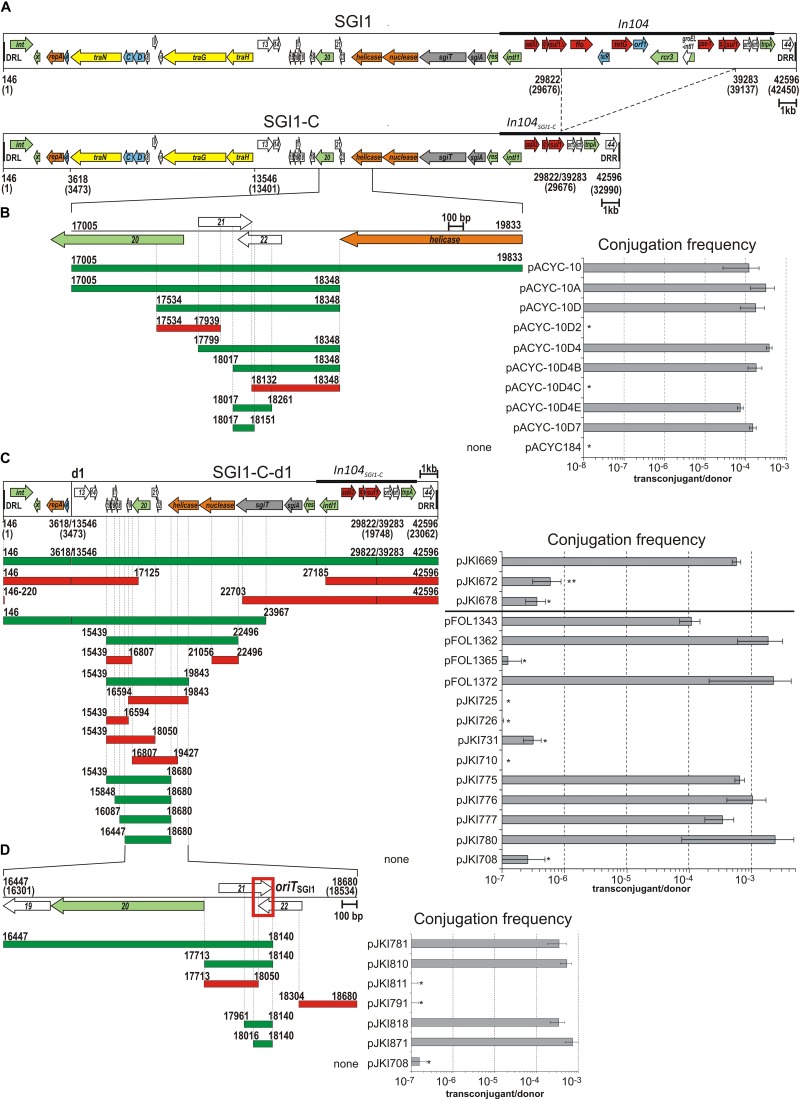
Identification of *oriT*_SGI1_. **(A)** Schematic map of SGI1 and SGI1-C. The annotated ORFs designated originally as S001-S044 are indicated by colored arrows: green – recombination, transposase; orange – replication; blue – transcription regulator; yellow – conjugation; gray – TA system; red – antibiotics resistance gene; white – unknown function. Names of genes with known function or identified homologs are indicated. Abbreviations: *x* - *xis*, *C* and *D* - *flhDC*_SGI1_, *sgiT* and *sgiA* – toxin and antitoxin gene of SGI1 TA system, *q*Δ – *qacE*Δ*1*, genes of unknown function are numbered according to their original numbering (e.g., “4” refers to S004, etc). Terminal direct repeats are shown as black boxes, In104 and its deletion derivative in SGI1-C are indicated. Coordinates are according to the published SGI1 sequence (AF261825) and refer to the ends of SGI1 (including DRs) and the endpoints of deletions led to the formation of SGI1-C and SGI1-C-d1 variants. The real coordinates of SGI1, SGI1-C and SGI1-C-d1 (taken into account of the missing A base from our SGI1 sequence at the 16338 bp position of AF261825) are indicated in brackets below the maps of SGI1 variants (5′-end of DRL was designated as the first position of SGI1). All maps are drawn to scale. **(B)** Identification of *oriT*_SGI1_ using the *S.* Agona 47SA97 (harboring R55 helper and the chromosomally integrated SGI1-C^Δ^*^int^*) donor and the *E. coli* BM14 recipient strains for mating. The color-coded horizontal bars with coordinates represent the cloned SGI1 regions in the pACYC184-derivative plasmids: green – mobilizable; red – not mobilizable. The graph shows the transfer frequency of the plasmids. Frequency data were calculated as transconjugant/donor titres in each figure. Asterisk indicates that the transfer frequency was below the detection limit ( < 10^-8^/donor). **(C)** Identification of mob_SGI1_ region in SGI1-C-d1 variant. Conjugation frequency of SGI1-C-d1 variant carried by a p15A based vector and its p15A-based subclones was measured in the presence of the helper plasmid R55, but without chromosomal SGI1. Horizontal bars indicate the SGI1-derived sequences present in the test plasmids. For mobilization of the constructs carrying the Sm^R^/Sp^R^ marker of SGI1-C-d1, TG1 donor and TG1Nal recipient, while for others, TG1Nal donor and TG2 recipient strains were used (separated by horizontal line). The asterisk indicates that the transfer frequency was close to or below the detection limits (<10^-7^/donor). The rare transconjugants obtained sporadically carried also the helper plasmid (co-transfer of the two plasmids was ∼100 %) suggesting an *oriT*_SGI1_-independent transfer mechanism (e.g., conduction, transfer of plasmids by cointegration with the helper). ^∗∗^In case of pJKI672, the Nal^R^Sm^R^ colonies obtained with a frequency of ∼6 × 10^-7^ did not contain the transferred plasmid, thus were not real transconjugants. Other symbols are as in panels **(A)** and **(B)**. **(D)** Identification of *oriT*_SGI1_ using mob_SGI1_-donor (harboring chromosomally integrated mob_SGI1_ and R55 helper) and TG2 recipient *E. coli* strains. The minimal fully mobilizable fragment designated as *oriT*_SGI1_ is indicated by red box, other symbols are as in panel **(B)**.

Among the few well-studied families of MGIs, the *Salmonella* genomic island 1 (SGI1) is one of the largest and most diverse IME family, which considerably contribute to spreading MDR and especially the resistance to critically important antibiotics such as extended-spectrum β-lactams or carbapenems. The prototype of SGI1 has been described in a multidrug resistant clone of *Salmonella enterica* serovar Typhimurium DT104 (Boyd et al., 2000), which emerged during the mid-1980s (Threlfall et al., 1994) and spread worldwide. The common multidrug resistance phenotype (ACSSuT) of this epidemic clone is conferred by the 42.4-kb SGI1, which contains 44 predicted open reading frames (ORFs) and carries a ca. 15 kb complex In4-type integron structure called In104 (Figure 1A). In104 is inserted near the 3′-end of the SGI1 backbone (Boyd et al., 2001) and flanked by 25-bp imperfect inverted repeats. In the prototype SGI1 this gene cluster contains two class 1 integron structures with gene cassettes *aadA2* and *bla*_CARB-2_ *(bla*_PSE-1_), respectively, other resistance genes (*tetA*(G) and *floR*) occurring independently of the integrons, IS elements (IS*CR3*, IS*6100*) and some additional genes of unknown function. Loss, gain or exchange of antibiotic resistance genes have occurred mainly in In104 by exchanges of resistance gene cassettes, homologous recombinations or IS-induced rearrangements, which led to the emergence of known SGI1 variants (SGI1-A to SGI1-Z12, SGI2) identified in numerous *S*. *enterica* serovars, *Proteus mirabilis* strains and *Morganella morganii* (Levings et al., 2005, 2008; Doublet et al., 2008; Siebor and Neuwirth, 2011, 2014; Schultz et al., 2017). Recently, SGI1-related elements have been described in several other species such as PGI1 and PGI2 in *P. mirabilis* and AGI1 in *Acinetobacter baumannii* (Siebor and Neuwirth, 2014; Hamidian et al., 2015). Other uncharacterized SGI1-related elements are found in *Aeromonas veronii*, *Vibrio cholerae* (Johnson et al., 2015), *Vibrio mimicus*, *Shewanella* and *Enterobacter* spp. (Supplementary Figure S1).

The SGI1-family islands are typical IMEs, as they integrate autonomously into or excise from the bacterial chromosome specifically at the 3′-end of *trmE* gene (formerly *thdF* and also called *mnmE*), but they do not encode all the genes necessary for their self-transfer, and require the conjugation system of plasmids of the incompatibility groups A or C for their horizontal transfer (Doublet et al., 2005, Daccord et al., 2010). The IncC family with hundreds of fully sequenced plasmids includes broad host range, single-copy conjugative plasmids implicated in MDR dissemination, while IncA group contains only one sequenced member, RA1, which has a backbone closely related to that of IncC plasmids, but proved to be compatible with them (Datta and Hedges, 1973; Ambrose et al., 2018). Their conjugative system, which is related to that of SXT/R391 ICEs (Wozniak et al., 2009; Poulin-Laprade et al., 2015), has been classified into the MOB_H12_ group (Garcillán-Barcia et al., 2009). SGI1 is mobilized by IncA and IncC (hereafter are referred as IncA/C) plasmids such as RA1, R55, R16a, IP40a, pVCR94 or pRMH760 (Douard et al., 2010; Kiss et al., 2012; Carraro et al., 2014; Harmer et al., 2016), however, other unrelated IMEs (e.g., MGI*Vmi1* of *V. mimicus*) have also been reported to utilize these plasmids as mobilization helpers (Carraro et al., 2014, 2016). Expression of the conjugation apparatus of IncA/C plasmids are controlled by the FlhDC-family master activator, AcaCD (Carraro et al., 2014), which is required for the transfer of both the plasmid and the mobilized IMEs (Carraro et al., 2014; Kiss et al., 2015). The plasmid-encoded AcaCD triggers the excision of SGI1 through the activation of expression of the SGI1-encoded recombination directionality factor Xis (Carraro et al., 2014; Kiss et al., 2015), but has no effect on the expression of the site-specific recombinase Int (Kiss et al., 2015) required for both the excision and integration (Doublet et al., 2005). After SGI1 transfer, the island efficiently integrates into the *attB* site in *trmE*.

Recent studies have indicated a more complex crosstalk between SGI1 and IncA/C plasmids. In addition to the *xis* promoter, four other AcaCD-responsive promoters have been identified on SGI1 (Carraro et al., 2014; Murányi et al., 2016). Moreover, the island also encodes its own functional FlhDC-family regulator, FlhDC_SGI1_ (Kiss et al., 2015). AcaCD and the closely related FlhDC_SGI1_ can activate the AcaCD-responsive promoters on both SGI1 and the conjugation regulon of IncA/C plasmids (Murányi et al., 2016). The SGI1-encoded transfer proteins TraN, TraG, and TraH, whose expression is also under AcaCD-control, interact with the plasmid encoded homologs and promote SGI1 transfer at the expense of the helper plasmid (Carraro et al., 2017). Furthermore, SGI1 and IncA/C plasmids encode for incompatibility functions impeding stable co-habitation of the partners in the same host (Harmer et al., 2016; Huguet et al., 2016). Destabilization of SGI1 by IncA/C plasmids seems to be based on triggering of SGI1 excision by AcaCD (Kiss et al., 2015), however, the similar effect of SGI1 on the helper plasmids appear more complex and poorly understood (Harmer et al., 2016).

Even though many aspects of the SGI1-IncA/C dual system have been studied, the basic transfer functions of SGI1, i.e., the *oriT* and the putative self-encoded mobilization proteins remain undiscovered. The three SGI1-encoded *tra* genes, *traN*, *traG* and *traH*, have an important but not essential role in the transfer of the island (Carraro et al., 2017; Kiss et al., 2012). Other ORFs of SGI1 do not show sequence similarity to known *tra* genes and relaxases, including that of IncA/C plasmids, thus, the implication of an atypical relaxase in SGI1 transfer can not be excluded. Similarly, *oriT* of SGI1 can not be identified on the basis of sequence homology with *oriT* of IncA/C plasmids (Hegyi et al., 2017), despite the fact that the plasmid-encoded relaxase is required for efficient SGI1 transfer. In the present work, we identify the mobilization region of SGI1 carrying the *cis*-acting *oriT* sequence and two predicted ORFs encoding proteins that are essential for the conjugative transfer of the island. The minimal *oriT* sequence, *oriT*_SGI1_, has been characterized, and the importance of its inverted repeat (IR) motifs for the transfer was examined. Comparative analysis of SGI1-related elements confirmed that the mob_SGI1_ region is well conserved throughout the family, strengthening its importance in the horizontal transfer of SGI1. The predicted structure and possible function of the two transfer proteins identified have also been discussed.

## Materials and Methods

### Microbial Techniques and DNA Procedures

Relevant features of the bacterial strains and plasmids are listed in Supplementary Tables S1, S2, respectively. Bacterial strains were maintained at -80°C in LB broth containing 30% glycerol and were routinely grown in Luria-Bertani (LB) broth at 37°C supplemented with the appropriate antibiotics used at a final concentration as follows: ampicillin (Ap) 150 μg/ml, chloramphenicol (Cm) 20 μg/ml, kanamycin (Km) 30 μg/ml, spectinomycin (Sp) 50 μg/ml, streptomycin (Sm) 50 μg/ml, nalidixic acid (Nal) 20 μg/ml, gentamicin (Gm) 25 μg/ml, tetracycline (Tc) 10 μg/ml, sodium azide (Az) 500 μg/ml. Standard molecular biology procedures were carried out according to Sambrook et al. (1989). Detailed methodology of plasmid constructions is described in Supplementary Methods. Test/colony PCRs were performed using Dream Taq polymerase (Thermo Fisher Scientific) as previously described (Kiss et al., 2012). The amplicons for cloning were amplified with Phusion (Thermo Fisher Scientific) or Pwo (Roche) polymerases and sequenced on ABI Prism 3100 Genetic Analyzer (PerkinElmer). Oligonucleotide primers used in this work are listed in Supplementary Table S3. Primers annealing to SGI1 or R55 were designed according to the published sequence of SGI1 (GenBank: AF261825) and R55 (GenBank: JQ010984).

The prototype SGI1 carries five functional resistance genes, which makes difficult to set up mating or KO mutagenesis experiments, thus for mating assays, cloning or mutagenesis we used the Sm^R^/Sp^R^, Sul^R^ derivative, SGI1-C (Figure 1A), which was previously shown to have the same mobilization properties as SGI1 (Kiss et al., 2012).

The β-galactosidase assay was performed in five replicates according to Miller (1972) except that the cultures were grown at 37°C to an OD_600_ ∼0.3 in LB both and diluted at a ratio of 1:1 with Z buffer.

Primer extension reactions were carried out as described (Murányi et al., 2016) using the β-gal tester plasmids pMSZ1017 and pMSZ948 carrying the upstream regions of S019 and S020, respectively.

### Conjugation Assays

Deletion derivatives of SGI1-C-d1 in pJKI669 were generated by enzymatic digestion and re-ligation and introduced into Escherichia *coli* TG1/R55. Other smaller SGI1 backbone fragments were amplified by PCR, cloned into pJKI708 vector and transformed into TG1Nal/R55 strain. The resulting TG1 or TG1Nal donor strains harboring one of the test plasmids along with R55 helper were then used in standard mating assays with TG1Nal or TG2 recipients, respectively, as described previously (Kiss et al., 2012).

Mating with *S.* Agona donor strains were performed by mixing mid-log phase (OD_600_ = 0.5) cultures of the donor strains harboring SGI1 and R55 w/o pACYC184-derivative test plasmids and the sodium azide-resistant *E. coli* recipient strain BM14 in a ratio of 1:1. The mix was incubated overnight at 37°C without shaking and then cells were streaked on appropriate selective SS agar plates. Transconjugants were selected on sodium azide combined with antibiotics depending on the transferred element: SGI1 – Sm, R55 – Km and the pACYC184 derivatives – Tc. The transfer frequencies were calculated as the ratio of transconjugant and donor titers. Complementation assays for identification of promoter regions of S020 and S019 were carried out as described (Kiss et al., 2012), except that mating was done with TG90 recipient in 2YT plates for 6 h to reduce the growth of the donor over the recipient cells.

IncC plasmid R16a was used as helper plasmid in some mating assays due to its more suitable resistance markers. It has previously been shown to be as effective mobilizer of SGI1 as R55 (Douard et al., 2010). In mobilization tests of the S019 and S020 KO (Knock-Out) mutant or WT mob_SGI1_-containing plasmids (pJKI772, pJKI737, pFOL1372) or SGI1-C by R16a^WT^ or R16a^ΔTraI^ helper plasmids, the *E. coli* donor strains TG1Nal or TG1Nal::SGI1-C were used with TG90 recipient. Mobilization of SGI1-C^Δ^*^oriT^* and pMNI41 by R55^ΔTn*6187*^ helper plasmid was assayed by mating of *E. coli* TG1Nal donor and *E. coli* TG90 recipient starins. In case of low frequency transfer, rare transconjugants were detected by spreading 100 μl (instead of dropping 5 μl) of undiluted bacterial suspension obtained from the mating LB plates onto the appropriate selective plates.

### Targeted Gene KO Experiments

The PCR fragments for KO mutagenesis of S019, S020, S022 and *oriT*_SGI1_ were amplified from pKD3 template plasmid using primers delS019for-delS019rev, delS020for-delS020rev, delS022for-delS022rev, and deloriTfor-deloriTrev, respectively, (Supplementary Table S3). For promoting the gene replacement recombination between the targeted region and the respective KO PCR fragment, λ Red recombinase was expressed from the plasmid pKD46 or its Tc^R^ derivative plasmid pJKI842 using 1% L-arabinose as inductor at 30°C for 1.5 h. The Cm^R^ cassette was removed from the chromosomal KO alleles by expressing the FLP recombinase from the thermo-inducible expression plasmid pCP20 (Datsenko and Wanner, 2000), or by digestion with XbaI (present in FRT sites) followed by religation in the case of pFOL1372 derivatives. The plasmids having temperature-sensitive pSC101 replication system were maintained and cured at 30 and 42°C, respectively. In the resulting KO mutants, a short region was replaced with 83-bp sequence deriving from the PCR template plasmid pKD3. The replacements near the 5′-end of S019, S020, and S022 generated early stop codons in the ORFs.

The KO PCR fragment for the scarless deletion (Kolisnychenko et al., 2002) of Tn*6187* in R55 was amplified from pJKI1023 template plasmid using primers R55-dTn6187ABfor- R55-dTn6187Crev. After the λ Red-induced recombination/gene replacement, the Sm^R^ marker gene was eliminated from the resulting R55^ΔTn*6187*^::Sm^R^ plasmid by DSB-stimulated recombinational repair process induced by I-SceI cleavage. Expression of I-SceI from pMSZ934 was induced by 30 μg/ml chlortetracycline (cTc) O/N at 30°C in LB+Cm+Ap. The Sm^S^ clones were selected by replica plating and the scarless site was amplified and sequenced. To demonstrate that deletion of Tn*6187* did not affect the conjugation properties of R55, mating assay was conducted with TG1Nal donor and TG90Nal (Tc^R^) recipient strains, where R55 and its ΔTn*6187* derivative showed similar transfer frequencies (2.9 ± 1.0 × 10^-1^ and 2.5 ± 0.8 × 10^-1^, respectively).

### Construction of mob_SGI1_-Donor Strain

For chromosomal integration of the 16447–18680 bp SGI1 regions, the pLOFKm (Herrero et al., 1990) derivative pJKI796 was introduced into *E. coli* S17-1 λ*pir* strain, which allows the replication and transfer of R6K-based plasmids carrying the *oriT* of RK2. One of the S17-1 λ*pir*/pJKI796 transformant colonies was used as donor in a standard mating (Kiss et al., 2012) with TG1Nal recipient. The miniTn*10*::mob_SGI1_-Km^R^ chromosomal integrants were selected on LB+Nal+Km plates at 37°C O/N. Km^R^Nal^R^ transconjugants were streaked twice onto LB+Nal+Km plates and tested for Ap^S^ phenotype indicating the loss of plasmid backbone of pJKI796 (conservative transposition of the miniTn*10* unit). Then, R55 was transferred into the resulting strain from TG1/R55 in a standard mating. Transconjugants were selected on LB+Nal+Cm plates at 37 °C O/N, and streaked twice on LB+Nal+Cm plates to get rid of donor contamination.

### Bioinformatics

Sequence alignments were generated using the MultAlin interface^1^ (Corpet, 1988). Promoter motifs were predicted by BPROM^2^ (Solovyev and Salamov, 2011). For searching protein motifs MOTIF Search^3^ was used. Protein structure modeling was conducted using Phyre2^4^ (Kelley et al., 2015), Swiss-Model^5^ (Arnold et al., 2006) and PSIPRED^6^ (Buchan et al., 2013). All homology searches were performed with the NCBI BLAST and DELTA-BLAST server^7^. SGI1-related elements were identified via a nucleotide BLAST search in GenBank using SGI1 backbone as query sequence, which was generated from the reference SGI1 sequence AF261825 by deletion of the flanking non-SGI1 sequences of DRs and In104 region along with one copy of the 5-bp direct repeat (DR associated with integron IRi) delimiting the In104 gene cluster. Inverted repeat motifs were detected using mFold^8^ server (Zuker, 2003).

## Results

### Identification and Functional Analysis of the SGI1 Transfer Origin

#### Localization of the *oriT* Sequence in SGI1

To determine the location of *oriT*_SGI1_, 14 different segments covering almost the entire SGI1-C backbone (excluding In104) were cloned into the non-mobilizable low-copy number vector pACYC184. These plasmids (pACYC184-1 to pACYC184-14) were introduced into the *S. enterica* serovar Agona strain 47SA97SGI1^Δ^*^int^* harboring the IncC helper plasmid R55 and their mobilization were assessed in mating assays. Non-mobilizable chromosomal SGI1^Δ^*^int^* was used to provide all (yet unknown) necessary *trans* mobilization factors and to prevent the mobilization of pACYC184 derivatives along with SGI1 via homologous recombination-mediated cointegration. Unlike other plasmids, pACYC-10 carrying SGI1 sequence from position 17005 to 19833 bp (Figure 1B) proved to be mobilizable at a comparable frequency (1.2 ± 1.0 × 10^-4^) to that of the wild-type (WT) SGI1-C (SGI1 positions are given according to the GenBank entry AF261825 used as reference sequence). However, pACYC-10 could not be mobilized in the absence of SGI1^Δ^*^int^* in the donor strain (transfer frequency was below the 10^-8^ detection limit). This suggested that this SGI1 fragment (the region flanked by ORFs S020 and S023) carried at least the *cis*-acting *oriT* sequence of SGI1, while other mobilization functions provided *in trans* by SGI1^Δ^*^int^* were also required for mobilization of pACYC-10.

To determine the minimal *oriT*_SGI1_, the fragment carried by pACYC-10 was progressively reduced in size and assessed for mobilization in similar assays (Figure 1B). The shortest mobilizable clone was pACYC-10D7 carrying a 135-bp SGI1 sequence extending from 18017 to 18151 bp, indicating that the fully functional *oriT*_SGI1_ overlaps the 3′-end of the predicted ORFs S021 and S022. The analysis of 10 transconjugants from each independent positive experiment confirmed the presence of the pACYC derivatives and the absence of the IncC helper plasmid, implying their conjugative mobilization *in trans*.

#### Minimal Mobilizable Region of SGI1

In parallel with the above approach, an alternative method was also applied to determine the entire region carrying all *cis*- and *trans*-acting mobilization functions of SGI1. The ca. 23 kb deletion derivative of SGI1-C (designated SGI1-C-d1, Figure 1C), which was previously shown to be mobilizable by R55 (Kiss et al., 2012) was cloned into a non-mobilizable p15A-based vector (plasmid pJKI669). Deletion derivatives and subclones of pJKI669 were used in mating assays in absence of chromosomal SGI1 in the donor strain. Only the plasmid constructs containing the intact S019-S022 region (position 16447 to 18680 bp) proved to be mobilizable *in trans* by R55 at a frequency comparable to the transfer of SGI1-C-d1 from pJKI669 (5.74 ± 0.93 × 10^-4^, Figure 1C). The shortest mobilizable construct, pJKI780, contained a 2.2 kb insert spanning SGI1 from the end of S019 to that of S023. Plasmids in which S019 (pJKI710, pJKI725), S020 (pJKI710, pJKI726) or S022 (pJKI731) were partially or entirely missing could not be mobilized. These results suggested that the 2.2 kb region cloned in pJKI780, hereafter called as mob_SGI1_, carries not only the *oriT*_SGI1_, but also all genes that are indispensable for SGI1 mobilization.

#### Identification of the Minimal *oriT*_SGI1_

Since further reduction of cloned mob_SGI1_ region impaired the transfer of the test plasmid, exact localization of *oriT*_SGI1_ and the determination of the minimal functional *oriT*_SGI1_ region required a helper strain which provides all mobilization factors *in trans* similarly, to the *S.* Agona strain 47SA97SGI1^Δ^*^int^* applied previously for mobilization of pACYC184-derivatives. Thus, a donor strain containing the chromosomally integrated mob_SGI1_ region was constructed. The 16447–18680 bp mob_SGI1_ region was integrated into the chromosome of *E. coli* strain TG1Nal using a mini-Tn*10* transposon (Herrero et al., 1990), and then the helper plasmid R55 was introduced. The resulting “mob_SGI1_-donor” strain was used to test the mobilization of plasmid subclones carrying progressively shortened fragments of mob_SGI1_ region by similar method applied above (see Figure 1C). The smallest SGI1 fragment permitting the mobilization of the test plasmid corresponded to positions 17961–18140 of SGI1 (pJKI818, Figure 1D). Based on the alternative methods (Figure 1B,D), we identified a 125-bp sequence corresponding to the 18016–18140 bp fragment of SGI1 (deduced from the overlap of inserts in pJKI818 and pACYC-10D7) as the minimal fully active *oriT*_SGI1_ (pJKI871, Figure 1D), which is localized in the 3′-end of ORF S021 including the overlapping part of S022.

#### Deletion of *oriT*_SGI1_ Causes Transfer-Deficiency

To further examine the *oriT*_SGI1_, we knocked out this segment from SGI1-C using the one-step gene inactivation method. In the conjugation assays, mobilization of SGI1-C^Δ^*^oriT^* and a p15A-based plasmid carrying the 125-bp *oriT*_SGI1_ sequence (pMNI41) was assessed. For this assay, the R55^ΔTn*6187*^ helper plasmid was used due to its reduced antibiotic resistance spectrum (Flo^R^/Cm^R^, Sul^R^). While the SGI1-C^Δ^*^oriT^* mutant proved to be non-mobilizable (<7.3 ± 2.3 × 10^-8^), the plasmid pMNI41 was mobilized *in trans* at a frequency of 7.3 ± 2.9 × 10^-3^, which was comparable to the transfer rate of pJKI871 also containing *oriT*_SGI1_ (Figure 1D). This result indicated that deletion of *oriT*_SGI1_ sequence abolished the SGI1 transfer, while the functions required *in trans* for mobilization of *oriT*_SGI1_-carrying plasmid were not affected.

#### Functional Analysis of Mutations Affecting IR Motifs in *oriT*_SGI1_

**FIGURE 2:**
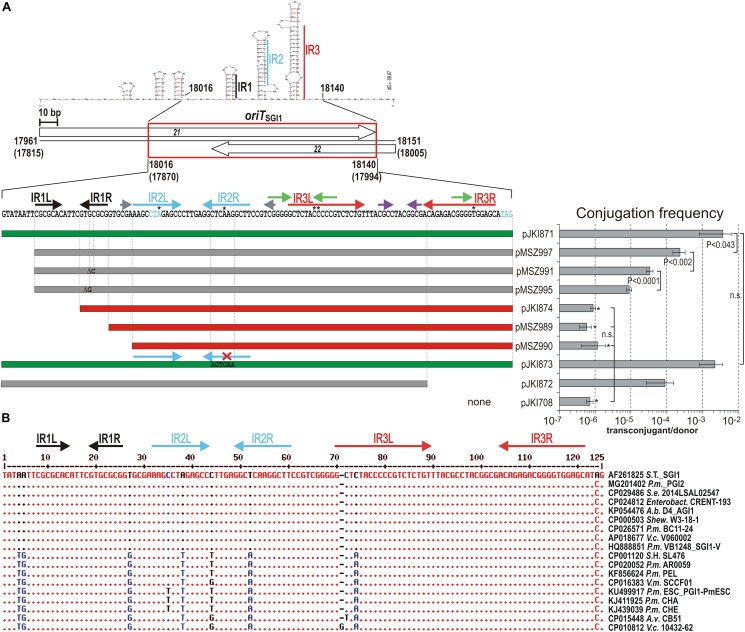
Mutation and comparative analysis of *oriT*_SGI1_. **(A)** Functional analysis of *oriT*_SGI1_. The location of fully functional *oriT* is indicated by red box in the schematic map. The SGI1 coordinates are indicated as in Figure 1. Repeated motifs are shown above the sequence by color-coded arrows. Left and right arms of the inverted repeats longer than 6 bp are designated as IR1/2/3L and R, respectively. Asterisks above the sequence indicate mismatching bases in the IRs. The potential secondary structure of the region and the stem-forming IRs are also shown. Shortened or mutated subclones of *oriT* are represented by color-coded horizontal bars: green – wt transfer rate; gray – reduced transfer rate; red – not mobilizable. Single base deletions and the sequence of mutagenized IR2R are indicated in the respective bars. The transfer frequencies of the pJKI708-derivative constructs were measured using the mob_SGI1_-donor and TG2 recipient strains. Asterisk indicates that the transfer frequency was close to or below the detection limit. The rare Tc^R^Sm^R^ colonies carried also the helper plasmid (co-transfer was ∼100 %) indicating that they did not result from *in trans* mobilization of the test plasmids. In all statistical evaluations paired t-test was used to calculate the significance of the differences. n.s. – not significant, other symbols are as in Figure 1A. **(B)** Comparison of the homologous sequences to *oriT*_SGI1_ in SGI1-related elements found in the GenBank database. The alignment shows the sequence divergence in the *oriT*_SGI1_ regions of 63 fully sequenced relatives of SGI1 (for the entire list see Supplementary Figure S1). The 17 *oriT* sequences having divergent positions are shown, while those identical to the reference sequence are represented by the respective region of the published SGI1 sequence AF261825. Short names of bacterial strains and the SGI1 variant/relative is indicated after the GenBank acc. number of the sequences. Abbreviations are as follows: *A.b*. – *Acinetobacter baumannii*; *A.v*. – *Aeromonas veronii*; *Enterobact*. - *Enterobacter* sp.; *P.m.* – *Proteus mirabilis*; *S*.*e*. – *Salmonella enterica; Shew*. – *Shewanella* sp.; *S*.T. – *Salmonella* Typhimurium; *S*.H. – *Salmonella* Heidelberg; *V.c*. – *Vibrio cholerae*; *V.m.* – *Vibrio mimicus.*

The *oriT*_SGI1_ sequence contains an array of inverted repeats (IRs, Figure 2A). For a more detailed analysis the *oriT* sequence, was further shortened or the repetitive motifs were mutated and the mobilization of the resulting *oriT*_SGI1_-derivatives was assayed using the mob_SGI1_ donor strain as described above. Mutations of the 7-bp perfect GC-rich inverse repeat IR1 resulted in significant reduction of the transfer frequency. Complete or partial deletion of IR1 entirely abolished the plasmid mobilization (pMSZ990, pMSZ989, and pJKI874; Figure 2A). Interestingly, deletion of the 7-bp AT rich tract located upstream of IR1 caused a 15-fold reduction of the transfer rate, which was further reduced by factors of 7 and 26 by the single base deletions in IR1R (compare pJKI871, pMSZ997, pMSZ995 and pMSZ991). Similarly, deletion of the right arm of the 19-bp imperfect repeat IR3 caused a 40-fold reduction of transfer rate (pJKI873 and pJKI872). In contrast, base changes introduced into the right arm of the 12-bp imperfect repeat IR2, abolishing the putative stem-loop structure, had no detectable effect on the transfer frequency. The two 6-bp GC-rich IRs located upstream of *oriT*_SGI1_ (17987–18017 bp, Figure 2A) seemed to have no or marginal role in *oriT* function, as their absence did not reduce the transfer frequency (compare pJKI818 and pJKI871, Figure 1D). The 125-bp *oriT*_SGI1_ sequence proved to be well conserved (95–100% identity) among the 63 SGI1-related IMEs found in public databases as only 11 divergent positions can be found in 17 elements (Figure 2B). These results confirmed that the fully functional *oriT*_SGI1_ is located between 18016 and 18140 bp of SGI1.

### SGI1 Encodes Two Essential Mobilization Proteins

#### Identification of ORFs Required for SGI1 Mobilization

The mob_SGI1_ region containing 4 annotated ORFs S019, S020, S022, and S021 (latter is encoded on the upper strand) proved to carry all *cis-* and *trans*-acting elements that are required for mobilization of SGI1 (pJKI780 in Figure 1C or the mob_SGI1_-donor strain). Mobilization of plasmid pMNI41 from the TG1Nal::SGI1-C^Δ^*^oriT^* donor strain, where both S021 and S022 are partially deleted due to the Δ*oriT* mutation suggested that the proteins encoded by the ORFs S021 and S022 are not required for *in trans* mobilization initiated at *oriT*_SGI1_. The Δ*oriT* mutation removes 40 and 29 amino acid (AA) residues from the C-terminus of the putative S021 and S022 proteins, respectively, which makes improbable that these proteins (if expressed at all) are still functional. To assess their role, independent KO mutations were generated in the 5′-end of both ORFs in mob_SGI1_-containing plasmids. As expected, transfer rates of both mutants were similar to the respective WT plasmid (2.7 ± 0.54 × 10^-3^ vs. 2.0 ± 0.32 × 10^-3^ for the S021 frameshift mutant pMSZ957/WT pMSZ949 and 6.1 ± 2.7 × 10^-4^ vs. 8.0 ± 1.7 × 10^-4^ for the S022 KO mutant pJKI774/WT pFOL1372) confirming that ORFs S021 and S022 do not encode proteins that are required for *in trans* mobilization of SGI1.

**FIGURE 3:**
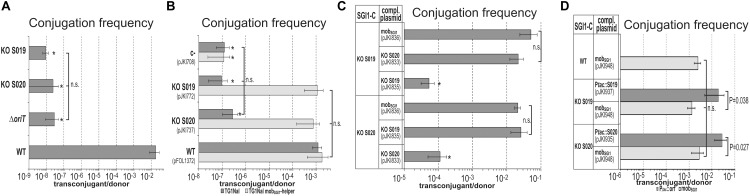
Mutation and complementation analysis of S019 and S020. **(A)** Transfer frequency of SGI1-C KO mutants. SGI1-C^WT^ and the KO mutants were mobilized by the R55^ΔTn*6187*^ helper plasmid from TG1Nal strain into TG2 recipient. The asterisk indicates that the transfer frequency was close to the detection limit. In case of SGI1-C^Δ^*^oriT^* the high rate of co-transfer with R55 among the sporadically occurring transconjugants refers to an *oriT*_SGI1_-independent way, while transconjugants of KO S019 and S020 SGI1-C mutants did not contain the helper plasmid and might derive via a yet unexplored way of *in trans* mobilization or lost the helper plasmid after conduction. **(B)** Transfer frequency of S019 and S020 KO mutant mob_SGI1_ regions cloned in pJKI708. In the mating assays TG1Nal/R55 or TG1Nal::mob_SGI1_/R55 strains containing the KO mutant or control plasmids were used as donor strains and TG2 was the recipient. Basal level of plasmid transfer (including the negative control plasmid pJKI708) indicated by asterisk did not result from regular *in trans* mobilization (high co-transfer rate with R55). **(C)** *Trans*-complementation of SGI1-C^ΔS019^ and SGI1-C^ΔS020^ by the respective KO mutant mob_SGI1_ regions. Donor *E. coli* strains TG1Nal::SGI1-C^ΔS019^ and TG1Nal::SGI1-C^ΔS020^ carried the helper plasmid R16a and one of the complementing plasmids containing the WT, KO S019 or KO S020 mob_SGI1_ region (pJKI836, pJKI835or pJKI833, respectively). ^∗^ Transconjugant frequency was below or around the detection limit. The relatively high detection limit was due to the lower donor titers compared to other matings (1.2–8.2 × 10^6^ CFU/ml instead of the general 2–5 × 10^9^ CFU/ml) in this experimental setup that was caused by the presence of two plasmids along with SGI1 in the donor strains. **(D)** *Trans*-complementation of SGI1-C^ΔS019^ and SGI1-C^ΔS020^ by expression of S019 or S020 proteins. Mating assays were performed using the recipient strain TG2 and donor strains TG1Nal::SGI1-C^WT^, TG1Nal::SGI1-C^ΔS019^, or TG1Nal::SGI1-C^ΔS020^ harboring the helper plasmid R16a and one of the complementing plasmids: pJKI948 contained the mob_SGI1_ region, while pJKI937 and pJKI935 expressed S019 and S020, respectively, from P*_tac_* promoter. Protein expression was driven by leakage of the *tac* promoter (without IPTG induction).

On the other hand, KO mutations of S019 and S020 had a deleterious effect on SGI1 conjugation, as the transfer frequency of both mutants dropped below 10^-7^ like that of the SGI1-C^Δ^*^oriT^* mutant (Figure 3A). Interestingly, few transconjugant colonies could be obtained with a frequency of 0.9–2.9 × 10^-8^. Phenotypic and PCR analyses showed that the rare SGI1-C^ΔS020^ and SGI1-C^ΔS019^ transconjugants carried SGI1 predominantly alone, unlike SGI1-C^Δ^*^oriT^* transconjugants, where the residual SGI1 transfer was tightly coupled to the conjugation of the helper plasmid. The absence of helper plasmid in the transconjugants suggested that the KO S020 and KO S019 mutants were *trans*-mobilized, albeit at a very low frequency. Similar result was obtained when the same KO mutations were introduced into the mob_SGI1_ region cloned in a p15A-based plasmid: the mobilization rates of the resulting KO S020 (pJKI737) and KO S019 (pJKI772) plasmids were 4 orders of magnitude lower than that of the WT control (pFOL1372) and were similar to the negative control (pJKI708). Using the mob_SGI1_-donor strain, WT transfer rates were observed with both KO mutant plasmids, indicating the effective *trans*-complementation by the chromosomally integrated mob_SGI1_ region (Figure 3B).

#### Complementation of S020 and S019 KO Mutants

ORFs S020 and S019 are separated by a single TTG codon (beyond the stop codon of S020). To assess whether the two ORFs are translated into a single, biologically active fusion protein by a read-through mechanism or into two independent proteins, SGI1-C^ΔS020^ and SGI1-C^ΔS019^ mutants were complemented with plasmids carrying S020 and S019 KO mutant mob_SGI1_ regions. While neither SGI1-C mutant could be complemented by the respective KO mutant mob_SGI1_ region, SGI1-C^ΔS020^ was complemented by S019 KO mob_SGI1_ region and *vice versa* as efficiently as was complemented by the WT mob_SGI1_ region (Figure 3C). This indicated that the two ORFs are translated into independent functional proteins.

*Trans* complementation of S020 and S019 KO mutant SGI1 was also carried out using expression vectors, which produced the two proteins under the control of P*_tac_* promoter. These plasmids (pJKI935 and pJKI937 expressing S020 and S019, respectively) were used to complement the TG1Nal::SGI1-C^ΔS020^ and TG1Nal::SGI1-C^ΔS019^ donor strains harboring the IncC helper plasmid R16a. In similar mating assays used previously, both expression plasmids efficiently complemented the respective SGI1 mutants (Figure 3D). About tenfold higher rates of SGI1-transfer were observed with the non-mobilizable expression plasmids compared to the mobilizable control pJKI948, probably due to the lack of competition between SGI1 and the complementing plasmids. These data confirmed that ORFs S020 and S019 encode two essential transfer proteins, thus, they have been designated as *mpsA* and *mpsB* (mobilization protein of SGI1), respectively.

### The Relaxase of IncC Helper Plasmid Is Not Essential for SGI1 Mobilization, but Increases Its Efficiency

**FIGURE 4:**
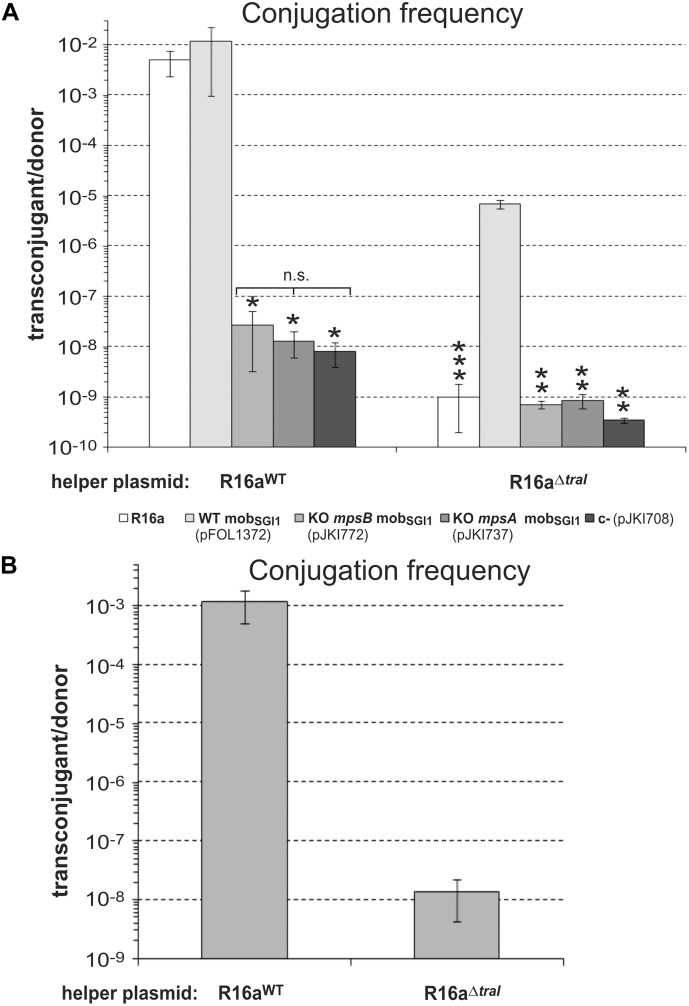
The role of *mpsA*, *mpsB* and the helper encoded relaxase *traI* in SGI1 mobilization. **(A)** Transfer frequency of the p15A-based plasmids, containing WT (pFOL1372), KO *mpsB* (KO S019, pJKI772) and KO *mpsA* (KO S020, pJKI737) mob_SGI1_ regions was assayed in the presence of the WT or relaxase KO mutant helper plasmid R16a. For mating, TG1Nal donor and TG90 recipient *E. coli* strains were used. The bars show means of 4 independent experiments except those representing the transfer rate of the helper plasmids. Since the conjugation frequency of the helper plasmids was the same independently of the test plasmids, the transfer rate of the helper plasmids R16a^WT^ and R16a^ΔTraI^ correspond to the mean of their transfer frequencies in the 4 different settings that have been repeated 4 times. ^∗^Transconjugants indicated by an asterisk carried also the helper plasmid (co-transfer was ∼100 %) suggesting that the transfer of test plasmids did not derive from regular *trans*-mobilization. ^∗∗^Transfer frequency was below the detection limit (transconjugants were not obtained). ^∗∗∗^Transfer frequency of R16a^ΔTraI^ was close to the detection limit, 6 transconjugant colonies were obtained from five independent experiments (mean frequency was ≤ 1.9 × 10^-9^). These colonies did not contain test plasmid. **(B)** Mobilization of SGI1-C^WT^ by the helper plasmids R16a^WT^ and R16a^ΔTraI^ from TG1Nal::SGI1-C donor into TG90 recipient strain.

SGI1 exploits the transfer machinery of IncA/C plasmids in multiple manners (Kiss et al., 2015; Carraro et al., 2017), however, the role of the relaxase encoded by the *traI* gene of IncA/C plasmids in SGI1 mobilization has not yet been analyzed. To assess the possible cooperation between the relaxase and MpsAB proteins in mobilization of SGI1, p15A-based plasmids carrying the WT or *mpsA* or *mpsB* KO mutant mob_SGI1_ regions were compared in mating assays in the presence of the WT or the relaxase KO mutant helper plasmid, R16a^WT^ and R16a^ΔTraI^, respectively. R16a^WT^ efficiently mobilized the plasmid containing the WT mob_SGI1_ region, while the two KO mutants (Δ*mpsA* and Δ*mpsB)* showed similar transfer rates to the negative control (Figure 4A). These data were in accordance with the previous results obtained with the helper plasmid R55 (Figure 3B) confirming that both *mpsA* and *mpsB* genes are required for transfer of the mob_SGI1_ region. Interestingly, the helper plasmid R16a^ΔTraI^, which is unable to conjugate, mobilized the plasmid containing WT mob_SGI1_ region at a frequency of 6.9 ± 1.4 × 10^-6^, although with ca. 3 logs lower frequency compared to R16a^WT^ (Figure 4A). Analysis of 10 transconjugants from each independent experiment proved that all were devoid of the helper plasmid and derived from *trans*-mobilization of the mob_SGI1_ plasmid. Moreover, no transconjugant at all was obtained in absence of either *mpsA* or *mpsB*. The R16a^ΔTraI^ was also able to mobilize the chromosomally integrated SGI1-C, although with 5 logs lower efficiency compared to R16a^WT^ (Figure 4B). Transconjugants were proved to carry SGI1-C integrated at the *attB* site, and were devoid of the helper plasmid. These results indicate that *mpsAB* genes are indispensable, while the helper plasmid-encoded relaxase is also important for the efficient conjugative transfer initiated at *oriT*_SGI1_. However, mobilization rate of SGI1 remains detectable and significant in absence of the relaxase, suggesting that TraI provides important, but probably different functions in the initiation step of SGI1 transfer from the classical model.

### Identification of Promoter Regions Driving the Expression of *mpsAB* Genes

Based on the genetic context and the previous results, *mpsAB* genes were suspected to form a bicistronic operon. To localize the promoter region of this putative operon, the promoter activity of sequences located upstream of *mpsA* was measured in β-galactosidase assay. Two β-gal tester plasmids were constructed: the longer region fused to a promoterless *lacZ* gene extended from the start codon of *mpsA* to the end of S023 (pMSZ947), while the shorter one extended only to the end of the predicted ORF S022 (pMSZ948). The tester plasmids showed somewhat higher β-galactosidase activity (6.4 ± 1.5 and 4.2 ± 0.5 U, respectively) than the negative control plasmid pJKI990 (1.1 ± 0.4 U) suggesting that a weak promoter could drive the expression of the putative operon [for comparison P*_int_* of SGI1 produced ∼350 U β-galactosidase in the same assay (Kiss et al., 2015)].

**FIGURE 5:**
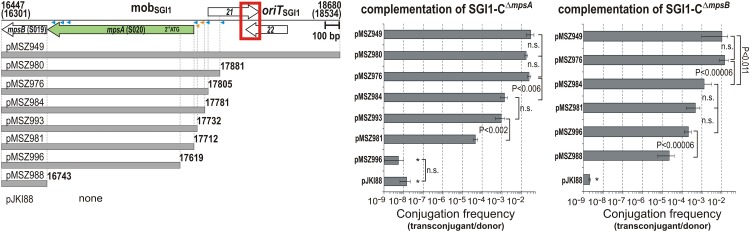
Identification of promoter regions driving expression of *mpsA* and *mpsB*. The fragments cloned into the complementing plasmids are shown below the schematic map of mob_SGI1_ region. Putative promoter elements predicted by BPROM or found by manual search are indicated by blue or yellow arrowheads, respectively. Other symbols are as in Figure 1B. Transfer frequency of SGI1-C^ΔS020^ and SGI1-C^ΔS019^ was measured in the presence of one of the complementing plasmids and the helper plasmid R55^ΔTn*6187*^. Plasmid pMSZ984 showed high st. deviations in three independent complementation assays carried out with 3–6 replicates with SGI1-C^ΔS019^, thus the complementation efficiency of this plasmid could not exactly be determined. However, mean values appeared similar to that of pMSZ981 and pMSZ996. ^∗^Transconjugants resulting from *oriT*_SGI1_-independent transfer pathway.

Then, a complementation method was applied to localize this weak promoter more exactly, where the mobilization rate of SGI1-C^Δ^*^mpsA^* and SGI1-C^Δ^*^mpsB^* mutants was examined in the presence of R55^ΔTn *6187*^ helper and p15A-based complementing plasmids, which produced MpsA and MpsB proteins *in trans* depending on the intactness of the promoter in the cloned mob_SGI1_ fragments. Both SGI1-C mutants could be complemented to the WT transfer rate achieved with the whole mob_SGI1_ region (pMSZ949) by the fragments carrying *mpsAB* genes and at least the non-coding region between *mpsA* and S021 (pMSZ976, pMSZ980, Figure 5). This suggested that the P*_mpsAB_* promoter is located in the ca. 100 bp region upstream of the *mpsA* start codon. Then, primer extension experiments were conducted as previously described (Murányi et al., 2016) to identify the transcriptional start site (TSS) of *mpsAB* using plasmid pMSZ948. However, in repeated attempts, it failed to detect TSS, which again suggested a low transcription level of *mpsAB*.

Thus, the complementation method was further applied to localize the active promoters by progressively shortening the upstream region of *mpsA* in the complementing plasmids. Removing of the distal 25 bp (pMSZ984) resulted in a ca. 20-fold and 10-fold decrease of the transfer rate of SGI1-C^Δ^*^mpsA^* and SGI1-C^Δ^*^mpsB^*, respectively, compared to the shortest fully active plasmid pMSZ976 (Figure 5). The similarly, decreasing complementation efficiency observed with the two KO mutants supported that the two ORFs are expressed from a common promoter located upstream of *mpsA*. Interestingly, pMSZ993, carrying only 20 bp segment upstream of the *mpsA* start codon, achieved relatively efficient complementation of SGI1-C^Δ^*^mpsA^* even though this segment was too short to contain an intact promoter. This raised the possibility that MpsA protein might be translated from the second, in-frame Met codon (17619 bp) instead of the originally identified start codon (17712 bp). This hypothesis was examined using two further complementing plasmids: pMSZ981 contained *mpsAB* without upstream sequences, while pMSZ996 carried a truncated *mpsA* beginning with the second Met codon. In both plasmids, the *rrnB* T1T2 terminators were inserted immediately upstream of the start codons to prevent any promoter activities from the plasmid backbone. The construct with truncated *mpsA* (pMSZ996) could not complement SGI1-C^Δ^*^mpsA^*, while pMSZ981 containing only the coding sequence of *mpsAB* without promoter region had a residual activity in complementation of SGI1-C^Δ^*^mpsA^* (ca. 540-fold reduction compared to the whole mob_SGI1_ region, pMSZ949, Figure 5). This result excluded that translation of MpsA protein starts from the in-frame Met codon and confirmed again that a very low level of MpsA expression is sufficient for complementation.

On the other hand, SGI1-C^Δ^*^mpsB^* could be complemented by both constructs at a comparable level observed with pMSZ984, indicating that *mpsB* can also be expressed independently from P*_mpsAB_*. Interestingly, like SGI1-C^Δ^*^mpsA^*, the transfer of SGI1-C^Δ^*^mpsB^* could be complemented by a construct, which contained only the coding sequence of *mpsB* separated from the plasmid backbone by *rrnB* terminators. To detect TSS of *mpsB*, an analogous plasmid to pMSZ948 was constructed, which carried the 227 bp upstream region of *mpsB* (the 3′ part of *mpsA*) fused to the promoterless *lacZ* gene (pMSZ1017), and primer extension assay was carried out, but without positive result. These suggested again that low level of MpsB can ensure the complementation of SGI1-C^Δ^*^mpsB^* (Figure 5). The complementation data indicated that the *mpsAB* genes can be transcribed into a bicistronic mRNA from a common promoter region P*_mpsAB_*, however, *mpsB* appeared to be expressed also from its own promoter P*_mpsB_* located in the 3′-end of *mpsA*.

The promoter search using BPROM server and additional thorough examination of the sequence revealed several putative promoters in the upstream regions of *mpsA* and *mpsB* (Figure 5). Their high divergence from the consensus of σ^70^ promoters and the low score values supported our earlier assumption that both the bicistronic *mpsAB* and *mpsB* mRNAs are synthesized at a low level from the two promoter regions.

## Discussion

The boom in bacterial genome sequencing in the last decade highly contributed to the discovery of numerous MGIs of the SGI1 and related families gathering IMEs implicated in the spread of antimicrobial resistance among several Gram-negative pathogens such as *S. enterica* serovars, *Proteus mirabilis* strains and a few other species. Despite the evidence that SGI1-related elements are specifically mobilized by conjugative IncA/C plasmids, their own mobilization components such as *oriT* and self-encoded conjugation proteins have not yet been identified.

In this study, the essential SGI1 conjugative mobilization functions were found to be clustered in the 2.2-kb mob_SGI1_ region (Figure 1C,D) carrying the *oriT*_SGI1_ and two essential genes (ORFs S020 and S019) named *mpsA and mpsB*. The fully active minimal *oriT* sequence has been localized in the vicinity of the *mpsAB* genes between 18016 and 18140 bp positions (GenBank accession AF261825), which includes the overlapping 3′ parts of two small predicted ORFs S021 and S022. Like many *oriT* regions identified to date, the *oriT*_SGI1_ also contains inverted repeat motifs. Three long IR motifs ( ≥ 7 bp, IRs1-3) have been found in the 125-bp minimal *oriT*_SGI1_. The most important one is the GC-rich 7-bp perfect IR1 (Figure 2A), suggesting its functional implication in binding relaxosome proteins and that the potential *nic*-site is probably located near this motif. Different methodologies have been successfully applied to identify *nic*-sites of several well-known conjugative plasmids and ICEs (Pansegrau et al., 1993; Llosa et al., 1995; Zechner et al., 1997; Núñez and De La Cruz, 2001; Varsaki et al., 2003; Lee and Grossman, 2007; Chen et al., 2010; Tsvetkova et al., 2010; Grove et al., 2013). However, the *nic*-site of one of the best studied ICEs, SXT, and the related IncA/C plasmids could not be defined yet [V. Burrus personal communication, (Hegyi et al., 2017)]. Although many efforts have been made to identify the *nic*-site in *oriT*_SGI1_ applying biochemical (primer extension, RACE PCR, *in vitro* nicking assay) and *in vivo* (interrupted mating) methods, all failed to give convincing results to date (data not shown).

In contrast to most *oriT* regions, *oriT*_SGI1_ is a GC-rich (60%) sequence compared to the SGI1 backbone (44%). Similar high GC-content (61%) can be seen in both *oriT*s of ICE*clc* element (Miyazaki and Van Der Meer, 2011), however, in this case the entire ICE has also high (62.5%) GC-content.

The largest part or the entire mob_SGI1_ region is present in all the 63 fully sequenced SGI1-related IMEs (Supplementary Figure S1). In the distant relatives (see bottom of Supplementary Figure S1), the 3′-end of *mpsB* homologs are more divergent and the distal part (ca. 210 bp at the 3′-end of mob_SGI1_ region) of the non-coding region adjacent to S023 along with ORF S023 are missing (e.g., in AGI1 or PGI2). The nucleotide identity of the mob_SGI1-_homolog regions to the corresponding SGI1 reference varies from 85 to 100%. The 125-bp *oriT*_SGI1_ sequence is highly conserved (95–100% identity) among the SGI1-related IMEs as only 11 divergent positions can be found in 17 out of the 63 elements (Figure 2B). Four and three divergent positions occur in the IR2L-space-IR2R region and in the 5′-end of IR3L, respectively, while the GC-rich 7-bp perfect IR1 is fully conserved. These observations are congruent with the conclusion of the experimental results, strengthening the importance of this region in SGI1 mobilization.

*OriT*_SGI1_ does not show striking similarity to that of IncA/C plasmids (*oriT*_A/C_), either in genetic context, sequence motifs or potential secondary structures (Supplementary Figures S2A,B). *OriT*_A/C_ locates in an intergenic region between divergent genes and overlaps the promoter of one of them, *mobI*, which encodes a plasmid-specific transfer factor that is indispensable for conjugation of IncA/C plasmids, but not required for SGI1 mobilization (Hegyi et al., 2017). Although *oriT*_A/C_ also contains several IRs, these are shorter and share no sequence similarity to those of *oriT*_SGI1_. Furthermore, the most important part of *oriT*_A/C_ is a 14-bp direct repeat motif, which has no similar counterparts in *oriT*_SGI1_. Other striking difference is the GC-content: while *oriT*_SGI1_ is rather a GC-rich sequence, *oriT*_A/C_ has a low GC content (37.4%). The only similarity with *oriT*_A/C_ as well as with *oriT*s of many other mobilizable elements is the location of *oriT*_SGI1_ in the vicinity of mobilization genes.

Two pivotal SGI1-encoded transfer genes, ORFs S020 and S019, have been identified and were renamed here as *mpsA* and *mpsB*, respectively. Unlike *mpsAB* genes, the two other small predicted ORFs of mob_SGI1_, S021 and S022, do not appear to encode proteins that are involved in transfer process. Complementation experiments showed that *mpsA* and *mpsB* separated by a single codon beyond the stop codon of *mspA* are translated into independent polypeptides, in part, from a bicistronic mRNA, however, MpsB protein can also be expressed from its own mRNA (Figure 3C,D, 5). Interestingly, the sequential reduction of the upstream region of *mpsA* caused gradual decrease in complementation efficiencies of the respective KO mutant, suggesting the presence of at least two different functional promoters in this region. The sequence-based prediction, which identified several putative promoters in the intergenic region between *mpsA* and S021 and in the 5′ part of S021 (Figure 5) supported this assumption. By BPROM prediction three potential promoters located upstream of *mpsB* (in the 3′ part of *mpsA*) were found approving that *mpsB* has also its own promoter region. All of these promoter-like sequences show low similarity to the σ^70^ consensus. The weak promoter activity observed in β-gal assays, the negative results of primer extension assays to detect TSSs and the fact that plasmid constructs carrying *mpsB* or *mpsAB* coding sequences without their upstream regions could complement the respective KO mutants indicated that very low amount of MpsA and MpsB proteins are sufficient to reach detectable or even WT transfer rates.

Despite the crucial role of *oriT*_SGI1_, MpsA and MpsB proteins in SGI1 mobilization, a very low transfer frequency was observed with each SGI1-C KO mutants (around 10^-8^/donor, see Figure 3A, 4A) and mob_SGI1_-plasmids (Figure 3B) suggesting that the helper plasmid is somehow able to mobilize SGI1 at a very low level independently of *cis*- and *trans*-acting mobilization factors of SGI1. In case of deletion of *oriT*_SGI1_, the majority (≥ 97%) of the rare transconjugants contained also the helper plasmid suggesting that the transfer of SGI1 or the test plasmids was not independent of the transfer of the helper plasmid. The most plausible explanation for this phenomenon is that co-integrates was formed with the helper plasmid. The low transfer rate and the lack of extensive homology between SGI1-C derivatives or mob_SGI1_-plasmids and the helper plasmids suggest the involvement of a *recA*-independent illegitimate recombination process. Further support for a *recA*-independent mechanism may be that similar transfer rates were observed with the *oriT*_SGI1_-free pJKI708 and its derivatives when the *recA^-^ E. coli* TG2 was used as donor strain (Figure 1C). A co-integrate based transfer mechanism has been reported previously (Hegyi et al., 2017), however, in that case the high transfer frequency was the consequence of an efficient site-specific recombination. The sporadically occurring helper-free transconjugants obtained with *mpsA* or *mpsB* KO mob_SGI1_-plasmids (≤3%, Figure 3B, 4A) and the generally helper-free SGI1-C^Δ^*^mpsA^* or SGI1-C^Δ^*^mpsB^* transconjugants (Figure 3A) may represent a yet unexplored way of inefficient mobilization by IncA/C plasmids in absence of the major transfer proteins MpsA and MpsB.

Both the homology searches and the structure predictions advocate that MpsA protein belongs to the Tyr-recombinase/integrase superfamily of DNA breaking-rejoining enzymes. The 126–225 AA tract of MpsA shows high homology to the DNA_BRE_C conserved C-terminal catalytic domain that is characteristic of site-specific integrases, bacterial recombinases XerD/C and type IB topoisomerases.

Phyre2 modeling confirmed that MpsA is related to the Tyr-recombinase family, as the best templates with known 3D-structure were XerH (Fold library id.: c5jjvA) and Cre recombinase (Fold library id.: c1ma7A) with 15–13% sequence identity, 91–94% coverage and 100% confidence. All the other hits showing > 90% coverage were also site-specific Tyr-recombinases (XerC,D; phage integrases). Similar result was obtained from Swiss-Model. Based on AA sequence alignments with related recombinases, the conserved catalytic nucleophile tyrosine residue was predicted at the C-terminus of MpsA protein (Y319). In addition to the Y319, DELTA-BLAST allowed to identify the R162, H247, R250, H251 in MpsA matching with the catalytic residues of the active sites of bacteriophage Hp1 Integrase (1AIH_A), Cre (2CRX_B), λ Integrase (1AE9_A) and IntI4 (2A3V_A). The *mpsA* gene appears to be well conserved among the 63 SGI1-related elements as only 19 show some sequence divergence compared to the reference SGI1 (99–87% identity). Although these base variations cause several changes in the predicted amino acid sequences (Supplementary Figure S3A), the only case when MpsA protein is apparently truncated and possibly inactive is the *Aeromonas veronii* CB51.

The *mpsB* gene encoding for the other essential mobilization factor of SGI1 is also similarly, conserved as only 17 of 63 elements show some DNA sequence variations in this gene (99–76% identity). These changes cause AA substitutions at 18 positions of the predicted MpsB proteins, however, truncation of the protein occurs only in *V. mimicus* SCCF01 and incomplete protein can be expressed in *A. veronii* CB51 (Supplementary Figure S3B). Using MotifFinder, the 98-AA-long MpsB protein was predicted to contain a phage integrase N-terminal SAM-4 like domain (PF13495, Pfam database), which was also supported by the best Phyre2 modeling based on the lambda integrase-like N-terminal domain template (Fold library id.: d1f44a1) and by Swiss-model where, following two eukaryotic proteins spectrin and plectin, the best hits were the N-terminal domains of XerH and Cre. Based on these data and PSIPRED secondary structure prediction (Buchan et al., 2013), MpsB appears to be an independent domain-like protein containing 4 α-helices of which the last three (20–82 AA) are related to a portion of the N-terminal core binding domain of lambda integrases (Swalla et al., 2003). This raises the possibility that MpsB is involved in DNA binding at *oriT*_SGI1_, however, a protein-recruiting function into the initiation complex cannot be ruled out either.

SGI1 exploits the transfer machinery of IncA/C plasmids in multiple manners (Kiss et al., 2015; Carraro et al., 2017), however, the role of the plasmid-borne relaxase TraI in SGI1 mobilization has not yet been analyzed. The mobilization assays using R16^ΔTraI^ helper plasmid provided an unexpected result. While conjugation of the helper plasmid appears strictly TraI-dependent, the WT mob_SGI1_-plasmid and SGI1-C proved to be mobilizable in absence of TraI (Figure 4), albeit with orders of magnitude less efficiently. Considering the striking difference between *oriT*_SGI1_ and *oriT*_A/C_ (Supplementary Figure S2), it is unlikely that similar relaxosome complexes are formed at these *oriT*s. The fact that SGI1 mobilization was found to be possible in absence of the helper-encoded relaxase suggests that other SGI1-encoded proteins ensure the essential relaxase functions, at least in part, in the SGI1 relaxosome at *oriT*_SGI1_.

The IncA/C relaxase TraI, although significantly increases the SGI1 transfer by a yet unknown mechanism, seems unable to mobilize *oriT*_SGI1_ in absence of MpsA or MpsB. Its role might be the unwinding of donor DNA or delivery of the relaxosome to T4SS by recruiting the coupling protein. Since the components of the initiation complex of IncA/C plasmids are only predictable through their homologies to those of well-studied model systems, the functions of TraI and involvement of other IncA/C-encoded proteins in the plasmid transfer and SGI1 mobilization need further investigations. In addition to the DNA nicking domain, other functional domains of relaxases, such as helicase domains (Llosa et al., 1996; Cheng et al., 2011; Alperi et al., 2013) and translocation signals, which are required for binding by the cognate coupling proteins and delivery of the relaxase-DNA complex to the T4SS (Lang et al., 2010), have been identified in several conjugation systems. Since the helicase domain seems to be the peculiarity of the IncW/N/F family relaxases (Llosa et al., 1996) and such domain have not yet been identified in TraI of IncA/C plasmids, the most possible way by which TraI can enhance SGI1 mobilization is to facilitate the transport of the relaxosome complex to or even through the T4SS. In addition to TraI, some conjugation systems require several auxiliary relaxosome components. In the case of IncPα plasmid RP4, TraJ protein is responsible for sequence-specific recognition of *oriT* and directing TraI to the origin of transfer (Ziegelin et al., 1989), TraH stabilizes the *oriT-*DNA complex through specific protein-protein interactions (Pansegrau et al., 1990) and TraK stimulates the relaxation by wrapping DNA near *oriT* (Ziegelin et al., 1992). In IncW plasmid R388, TrwA binds specifically to *oriT* and stimulates the ATPase activity of the coupling protein TrwB, which is indispensable to link the relaxosome complex to T4SS (Cabezón et al., 2015). Similar auxiliary factors are not yet known in the conjugative apparatus of IncA/C plasmids. Besides the structural components and the assembly factors of T4SS, only TraI and the indispensable transfer factor MobI have been described in IncA/C transfer. Although the exact function of MobI is not clear, it appears not to be involved in SGI1 mobilization (Hegyi et al., 2017).

Based on the results presented here we suggest that MpsA functions as an atypical relaxase of SGI1, which needs MpsB for binding and/or nicking *oriT*_SGI1_, but does not require TraI for the initial step of SGI1 relaxosome formation. This explanation is strengthened by the recent description of the atypical relaxase TcpM of plasmid pCW3 from *Clostridium perfringens* that appears related to tyrosine recombinases (our Phyre2 modeling showed the best homology of TcpM with XerH) without sequence similarity to known relaxases (Wisniewski et al., 2016). The fact that *mpsB* KO mutant mob_SGI1_-plasmid could not be mobilized even by a WT helper plasmid (Figure 4) suggests that MpsB rather has a role in the initiation of transfer (maybe in binding *oriT*_SGI1_ together with MpsA), than recruiting additional proteins like TraI. The relaxase TraI might be involved in unwinding the donor DNA or more possibly in the transport of the relaxosome to T4SS. The key elements and an accessory partner of the SGI1 relaxosome have been here identified, however, the understanding their exact role in the conjugative DNA processes needs further investigations.

## Data Availability

All datasets generated for this study are included in the manuscript and/or the Supplementary Files.

## Author Contributions

JK, BD, AC, and FO conceived the project. JK, MS, AH, GD, KP, BD, IN, and FO designed and carried out the experiments and analyzed the data. JK and BD performed the bioinformatic analyses, JK prepared the figures. JK, BD, AC, and AH wrote the manuscript. All the authors reviewed the manuscript.

## Conflict of Interest Statement

The authors declare that the research was conducted in the absence of any commercial or financial relationships that could be construed as a potential conflict of interest.

## Footnotes

1 http://multalin.toulouse.inra.fr/multalin/

2 https://omictools.com/bprom-tool

3 http://www.genome.jp/tools/motif/

4 http://www.sbg.bio.ic.ac.uk/phyre2/html/page.cgi?id=index

5 https://swissmodel.expasy.org/

6 http://bioinf.cs.ucl.ac.uk//psipred/

7 https://blast.ncbi.nlm.nih.gov/Blast.cgi

8 http://unafold.rna.albany.edu/?q=mfold/DNA-Folding-Form
