# Supplementary material for: Identification and Characterization of oriT and Two Mobilization Genes Required for Conjugative Transfer of Salmonella Genomic Island 1
Source: Front Microbiol. 2019 Mar 6;10:457. doi: 10.3389/fmicb.2019.00457 (PMC6414798; doi:10.3389/fmicb.2019.00457)
Supplement: Supplementary file 1 [file Data_Sheet_1.PDF]

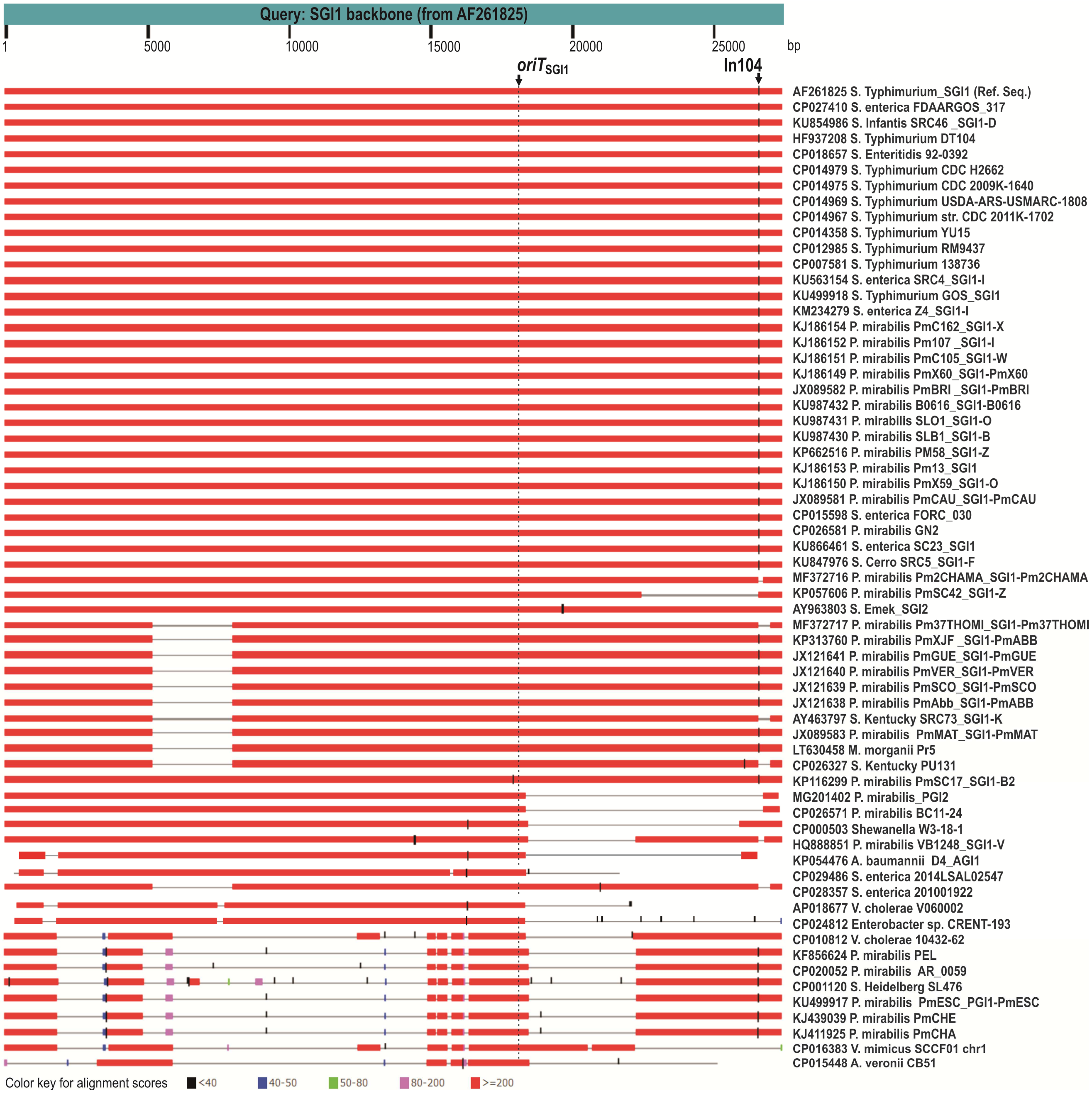

Figure S1. Alignment of 63 fully sequenced SGI1-related elements. SGI1 backbone sequence was generated by deletion of In104 region together with one copy of the 5-bp direct repeat delimiting the In104 gene cluster from the reference SGI1 sequence AF261825. The backbone was used as query sequence in nucleotide Blast search in GenBank database (data as of 07.06.2018). Accession number of the genome sequences, names of the species/strains and SGI1-related elements (if exist) are listed. Position of $oriT_{\mathrm{SGI1}}$ and In104 are indicated.

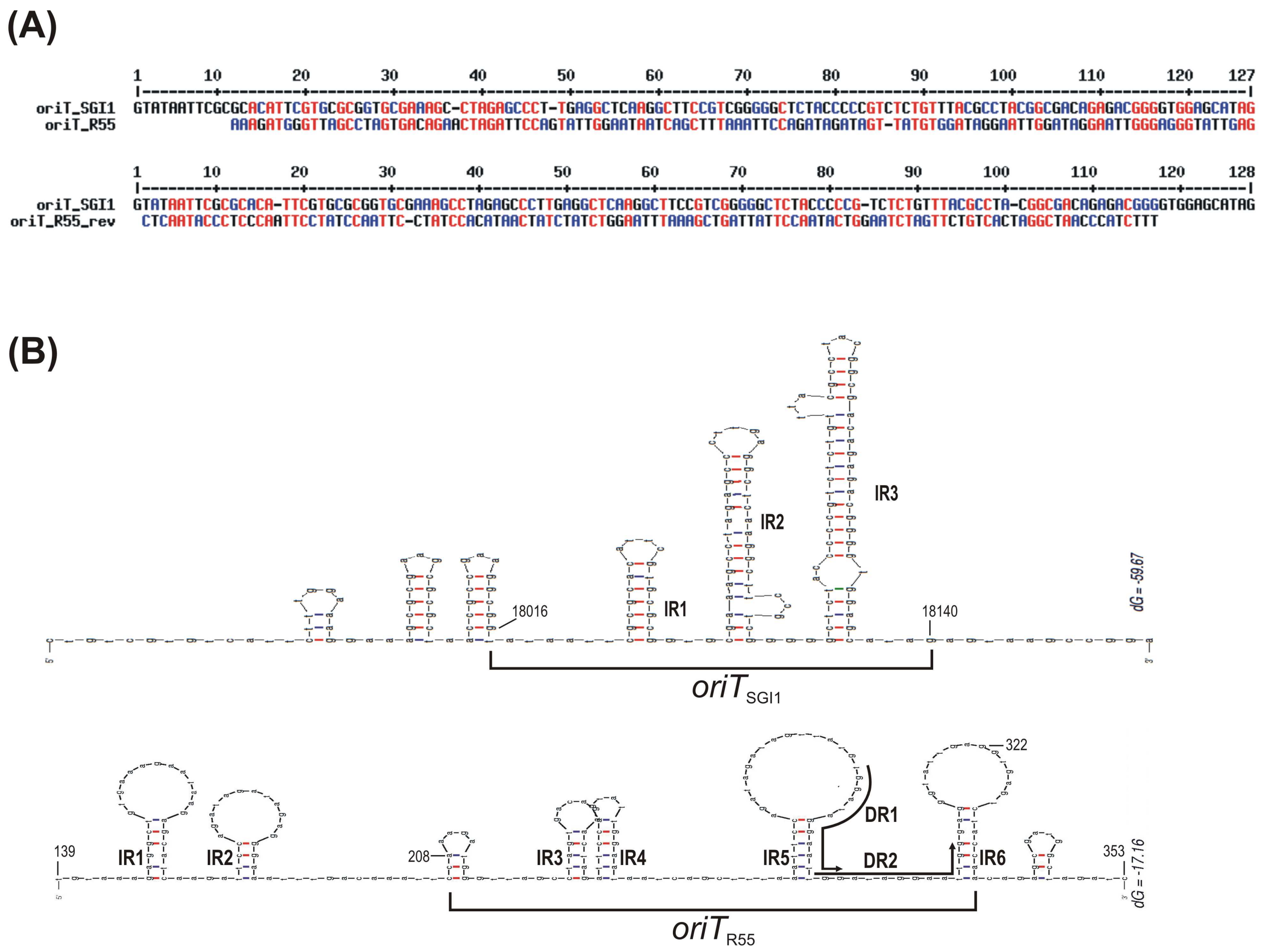

Figure S2. Comparison of *oriT*$_{SGI1}$ and *oriT*$_{A/C}$. (A) Sequence alignments of *oriT*$_{SGI1}$ and both strands of *oriT*$_{R55}$ (*oriT*$_{A/C}$). (B) Predicted secondary structure of *oriT*$_{SGI1}$ and *oriT*$_{R55}$. Coordinates are according to Genbank AF261825 and JQ010984, respectively.

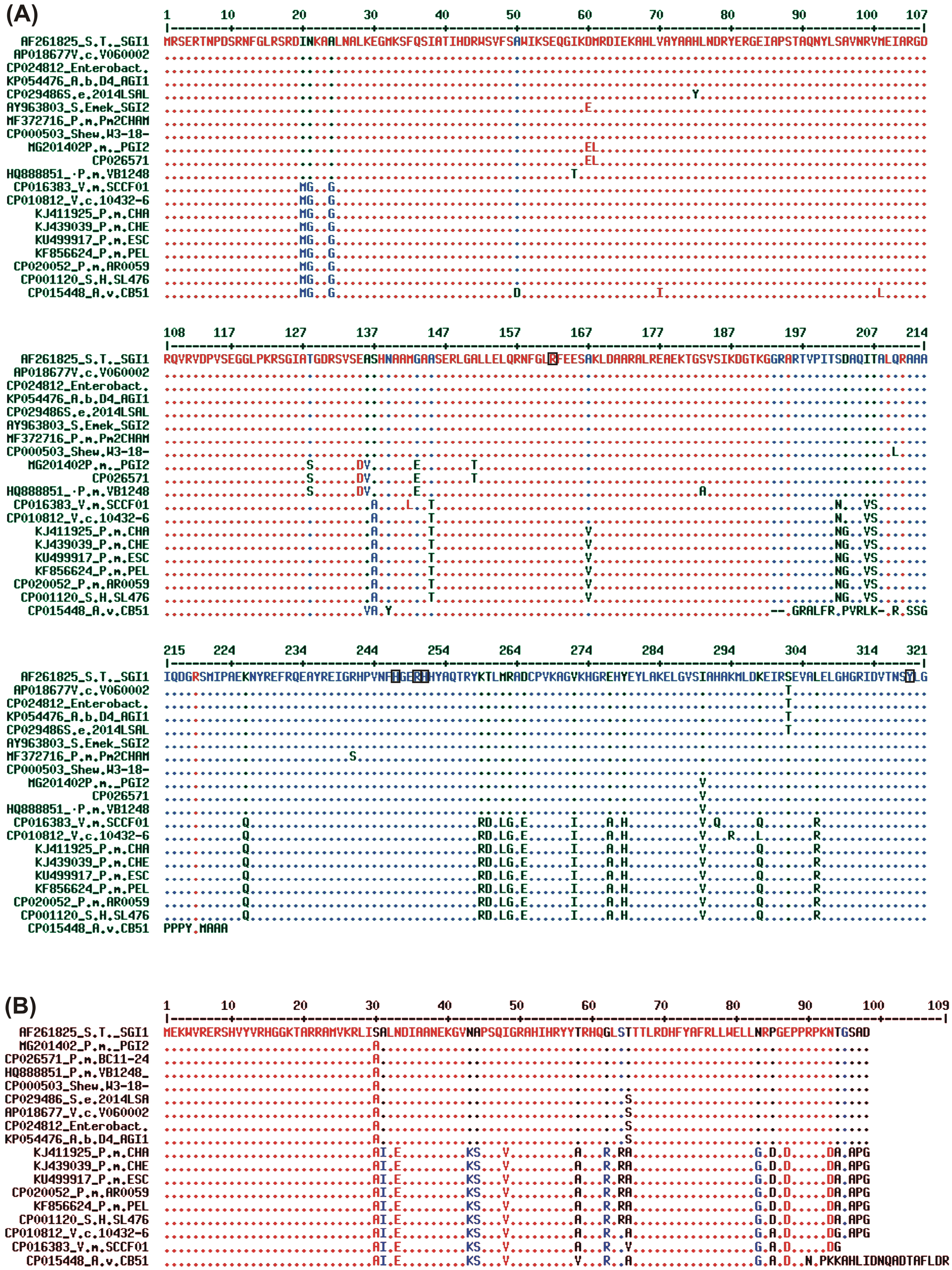

Figure S3. Alignments of the predicted MpsA and MpsB proteins encoded by the SGI1-related elements. The alignments show the amino acid divergences compared to the proteins encoded by the reference SGI1 sequence AF261825. The elements encoding identical MpsA and MpsB proteins with the reference are not shown (for the full list of the 63 SGI1-related elements, see Figure S1). (A) Alignment of MpsA homologs. The possible catalytic residues R162, H247, R250, H251 and Y319 predicted by DELTA-BLAST are boxed. (B) Alignment of MpsB homologs (data as of 07.06.2018).
