# Supplementary material for: Identification and Characterization of oriT and Two Mobilization Genes Required for Conjugative Transfer of Salmonella Genomic Island 1
Source: Front Microbiol. 2019 Mar 6;10:457. doi: 10.3389/fmicb.2019.00457 (PMC6414798; doi:10.3389/fmicb.2019.00457)
Supplement: Supplementary file 2 [file Data_Sheet_2.PDF]

Supplementary Methods. Construction of plasmids.

pACYC-1-pACYC-10D7 plasmids were all constructed as follows: The respective SGI1 segment was amplified with the appropriate primers (Table S3) carrying NcoI or EcoRI cleavage sites, respectively, at their 5' end (see below) and then the EcoRI-NcoI-digested PCR fragment was inserted into the *cat* gene of the EcoRI-NcoI-digested pACYC184.

pACYC-1: The 145-1351 bp region of SGI1was amplified with primers FwEcoRI1-RvNcoI1.

pACYC-2: The 1331-2324 bp region of SGI1was amplified with primers FwEcoRI2-RvNcoI2.

pACYC-3: The 2354-3874 bp region of SGI1was amplified with primers FwEcoRI3-RvNcoI3.

pACYC-4: The 3921-6536 bp region of SGI1was amplified with primers FwEcoRI4-RvNcoI4.

pACYC-5: The 6516-8851 bp region of SGI1was amplified with primers FwEcoRI5-RvNcoI5.

pACYC-6: The 8860-11754 bp region of SGI1was amplified with primers FwEcoRI6-RvNcoI6.

pACYC-7: The 11734-13434 bp region of SGI1was amplified with primers FwEcoRI7-RvNcoI7.

pACYC-8: The 13414-15164 bp region of SGI1was amplified with primers FwEcoRI8-RvNcoI8.

pACYC-9: The 15144-16913 bp region of SGI1was amplified with primers FwEcoRI9-RvNcoI9.

pACYC-10: The 17005-19833 bp region of SGI1was amplified with primers FwEcoRI10-RvNcoI10.

pACYC-11: The 19852-21930 bp region of SGI1was amplified with primers FwEcoRI11-RvNcoI11.

pACYC-12: The 21981-23590 bp region of SGI1was amplified with primers FwEcoRI12-RvNcoI12.

pACYC-13: The 23570-25250 bp region of SGI1was amplified with primers FwEcoRI13-RvNcoI13.

pACYC-14: The 25230-28141 bp region of SGI1was amplified with primers FwEcoRI14-RvNcoI14.

pACYC-10A: The 17005-18348 bp region of SGI1was amplified with primers FwEcoRI10-RvNcoI10A.

pACYC-10D: The 17534-18348 bp region of SGI1was amplified with primers FwEcoRI10D-RvNcoI10A.

pACYC-10D2: The 17534-17939 bp region of SGI1was amplified with primers FwEcoRI10D-RvNcoI10D2.

pACYC-10D4: The 17799-18348 bp region of SGI1was amplified with primers FwEcoRI10D3A-RvNcoI10A.

pACYC-10D4B: The 18017-18348 bp region of SGI1was amplified with primers FwEcoRI10D4B-RvNcoI10A.

pACYC-10D4C: The 18132-18348 bp region of SGI1was amplified with primers FwEcoRI10D4C-RvNcoI10A.

pACYC-10D4E: The 18017-18261 bp region of SGI1was amplified with primers FwEcoRI10D4B-RvNcoI10D4D.

pACYC-10D7: The 18017-18151 bp region of SGI1was amplified with primers FwEcoRI10D4B-RvNcoI10D3.

pFOL1343: The 2.08 kb BamHI fragment of pHP45Ω (Prentki and Krisch, 1984) carrying the Sm^R/Sp^R cassette was inserted into the BamHI site of MCS in pJKI671 (see below).

pFOL1362: The 7.05 kb PstI fragment of pJKI671 carrying SGI1 region from 15444 to 22496 bp (from 5' part of S015 to the 3' part of S025) was inserted into the PstI site of pJKI708.

pFOL1365 was generated from pFOL1362 by BglII digestion and religation, which removed the 16807-21056 bp region of SGI1.

pFOL1372 was generated by EcoRI digestion/religation of pFOL1362. The resulting plasmid contains 15439-22496 bp region of SGI1 (from 5' part of S015 to the 3' part of S023).

pJKI332 contains the Km^R gene of Tn*903* flanked by MCS-s assembled through multiple cloning steps as followings: The Km^R cassette was cloned from pUC4K (Vieira and Messing, 1982) with PstI into pGEM5Zf(+) (Promega) resulting in pJKI295, from which the Km^R cassette was transferred with SphI-SalI into the respective site of pEMBL19 (Dente et al., 1983) leading to pJKI298. Finally, the Km^R cassette was cloned from pJKI298 with HindIII-BamHI in pBluescript SK (Short et al., 1988) resulting in pJKI332.

pJKI669: The SGI1 *attB* ($attB_{Ec}$) was amplified from *E. coli* with attsgi1for-attsgi1rev primers (Kiss et al., 2012) and the amplicon was cloned into the XbaI site of pJKI88 (Kiss and Olasz, 1999), resulting in the pJKI627 trap vector (Kiss et al., 2012). The unique PstI site of pJK627 was eliminated by digestion with Klenow polymerase in absence of dNTPs leading to pJKI633. Plasmid pJKI669 was created by cloning SGI1-C variant carrying d1 deletion (spontaneous deletion of S005-S012 region of SGI1 backbone) with NotI from pJKI666::SGI28/1d1 (=pJKI666::SGI1-C-d1) (Kiss et al., 2012) into pJKI633. In the resulting plasmid, the 391 bp $attB_{Ec}$ fragment was replaced by the ca. 24 kb fragment carrying SGI1-C-d1 derivative integrated into the $attB_{St}$ site (Kiss et al., 2012).

pJKI671: Right part of SGI1-Cd1 was deleted by BamHI digestion/religation of pJKI669. The resulting plasmid contains DRL-S004 (1-3618 bp) and S013-S025 (36547-23967 bp) regions of SGI1.

pJKI672 is a deletion derivative of pJKI669 generated by BssHII digestion/religation. The plasmid contains DRL-S004 (1-3618 bp), S013-S019 (36547-17130 bp), Δ*intI1*-*sul1Δ* (27185-29822 bp) and *sul1*-DRR (39283-42596 bp) regions of SGI1.

pJKI678 was generated by MfeI digestion/religation of pJKI669. The plasmid contains DRL (1-220 bp), ΔS025-*sulΔ1* (22703-29822 bp) and *sul1*-DRR (39283-42596 bp) regions of SGI1.

pJKI710: The 2.62 kb BglII fragment of pJKI671 carrying SGI1 region from the 5' part of S020 to the 3' part of S023 was inserted into the BamHI site of pJKI708.

pJKI725: The 1150 bp PstI-SacI fragment of pFOL1372 carrying the SGI1 region from the 5' part of S015 to the 3' part of S019 was cut out and the remaining plasmid backbone was religated after digestion with Klenow polymerase in absence of dNTPs.

pJKI726: The 1150 bp PstI-SacI fragment of pFOL1372 carrying SGI1 region from the 5' part of S015 to the 3' part of S019 was ligated into the PstI-SacI site of pJKI708.

pJKI731: The 15431-18050 bp (S015-S021) region of SGI1 was amplified from pJKI669 with primers S020for1 and S020promrev. The amplicon was digested with EcoRI-PstI and ligated to the appropriate site of pJK708.

pJKI737: KO mutation in orf S020 was generated by the one-step gene inactivation method (Datsenko and Wanner, 2000) in pFOL1372. In the resulting plasmid, pJKI734, the 17571-17709 bp region in S020 of SGI1 was replaced with the Cm^R cassette amplified from pKD3. Then, the Cm^R gene was deleted with XbaI digestion and religation of the plasmid backbone leading to pJKI737. In the final sequence the replacement of the 139 bp tract following the start codon of S020 with 84 bp pKD3-derived sequence causes frameshift in orf S020.

pJKI772: KO mutation in orf S019 was generated by the one-step gene inactivation method in pFOL1372. In the resulting plasmid, pJKI769, the 16656-16739 bp region in S019 was replaced with the Cm^R cassette amplified from pKD3. The Cm^R gene was deleted with XbaI digestion and religation of the plasmid backbone leading to pJKI772. In the final sequence the replacement of the 84 bp tract following the 4th bp of S019 with 84 bp pKD3-derived sequence causes stop codon in orf S019 after the 6th codon.

pJKI774: KO mutation in orf S022 was generated by the one-step gene inactivation method in pFOL1372. In the resulting plasmid, pJKI771, the 18241-18323 bp region in S022 was replaced with the Cm^R cassette amplified from pKD3. The Cm^R gene was deleted with XbaI digestion and religation of the plasmid backbone leading to pJKI774. In the final sequence the replacement of the 83 bp tract of S022 with 84 bp pKD3-derived sequence causes stop codon in orf S022 after the 7th codon.

pJKI775: The 15431-18680 bp (S015-S022) region of SGI1 was amplified from pJKI669 with primers S020for1 and S022promrev. The amplicon was digested with EcoRI-PstI and ligated to the appropriate site of pJK708.

pJKI776: The 15849-18680 bp (S018-S022) region of SGI1 was amplified from pJKI669 with primers S020for2 and S022promrev. The amplicon was digested with EcoRI-PstI and ligated to the appropriate site of pJK708.

pJKI777: The 16087-18680 bp (S019-S022) region of SGI1 was amplified from pJKI669 with primers S020for3 and S022promrev. The amplicon was digested with EcoRI-PstI and ligated to the appropriate site of pJK708.

pJKI780: The 16447-18680 bp (S019-S022) region of SGI1 was amplified from pJKI669 with primers S020for4 and S022promrev. The amplicon was digested with EcoRI-PstI and ligated to the appropriate site of pJK708.

pJKI781: The 16447-18140 bp (S019-S021) region of SGI1 was amplified from pJKI669 with primers S020for4 and S021promrev. The amplicon was digested with EcoRI-PstI and ligated to the appropriate site of pJK708.

pJKI783: The EcoRI-PstI fragment of pJKI780 containing 16447-18680 bp (mob_{SGI1}) region of SGI1 was cloned in the appropriate site of pBluescript SK.

pJKI791: The 18304-18680 bp containing the upstream region of S022) was amplified from pJKI669 with primers S022promfor and S022promrev. The amplicon was digested with EcoRI-PstI and ligated to the appropriate site of pJK708.

pJKI796: The Km^R cassette from the mini-Tn*10* transposon of the R6K-based plasmid pLOFKm (Herrero et al., 1990) was deleted by NotI digestion and religation, leading to pJKI334. The Km^R cassette from pJKI332 was ligated into the unique SalI site of the MCS in pJKI783. The resulting plasmid, pJKI788, carried the 16447-18680 bp (S019-S022) region of SGI1 joined to the Km^R gene. This assembled region was then cloned into the NotI site between the IS*10* ends in pJKI334.

pJKI810: The 17713-18140 bp region of SGI1 containing the upstream sequence of S020 followed by S021 was amplified from pJKI669 with primers S021for and S021promrev. The amplicon was digested with EcoRI-PstI and ligated to the appropriate site of pJK708.

pJKI811: The 17713-18050 bp region of SGI1 containing the upstream sequence of S020 and the truncated S021 was amplified from pJKI669 with primers S021for and S020promrev. The amplicon was digested with EcoRI-PstI and ligated to the appropriate site of pJK708.

pJKI818: The 17961-18140 bp region of SGI1 containing the 3' part of S021 was amplified from pJKI669 with primers S021for2 and S021promrev. The amplicon was digested with EcoRI-PstI and ligated to the appropriate site of pJK708.

pJKI833, pJKI835 and pJKI836 was created from pJKI737, pJKI772 and pFOL1372, respectively, by inserting the Cm^R gene cassette of pAW302 (Stalder and Arber, 1989) as a HincII fragment into the ApaLI site of the Sm^R/Sp^R gene after filling in the protruding ends with Klenow polymerase.

pJKI842 was generated by cloning a Tc^R cassette into the Ap^R gene of pKD46. The Tc^R cassette containing the 4331-1314 bp region of pBR322 (Bolivar et al., 1977) was amplified using primers pBRTcPstfor – pBRTcPstrev. The amplicon was cloned via multiple steps into different MCSs of several cloning vectors, which resulted in the pEMBL19

derivative plasmid pJKI667, where the Tc^R cassette was bordered by complex MCSs. The Tc^R cassette was cloned as a SmaI fragment of pJKI667 into the PvuI site of the Ap^R gene in pKD46.

pJKI871: The 18016-18140 bp region (*oriT*) of SGI1 was amplified from pJKI669 with primers oriTfor and S021promrev. The amplicon was digested with EcoRI-PstI and ligated to the appropriate site of pJK708.

pJKI872: The 18016-18119 bp region (*oriT*ΔIR3R) of SGI1 was amplified from pJKI669 with primers oriTfor and oriTd2rev. The amplicon was digested with EcoRI-PstI and ligated to the appropriate site of pJK708.

pJKI873: The 18016-18082 bp region of SGI1 was amplified from pJKI669 with primers oriTfor and oriTIR2mutrev. The amplicon was used in a second PCR as megaprimer with S021promrev. The resulting amplicon then was digested with EcoRI-PstI and ligated to the appropriate site of pJK708.

pJKI874: The 18035-18140 bp region (*oriT*ΔIR1L) of SGI1 was amplified from pJKI669 with primers oriTd1for and S021promrev. The amplicon was digested with EcoRI-PstI and ligated to the appropriate site of pJK708.

pJKI876: The SGI1 region from 17712 to 16447 bp (S020-S019) was amplified with primers S020Ndefor and sgi_orf019rev, then the amplicon was cloned into the SmaI site of pBluescript SK and sequenced.

pJKI935: Orf S020 was amplified with primers sgi_orf020for and sgi_S020rev, ligated into the SmaI site of pBluescript SK and sequenced (pJKI922), then orf S020 was transferred from pJKI922 into the NdeI-BamHI site of pJKI391 resulting in the expression vector pJKI927. Finally, the Cm^R gene cassette of pAW302 (Stalder and Arber, 1989) was inserted into the SmaI site of the Km^R gene in pJKI927 as a HincII fragment.

pJKI937: The 16447-16743 bp region (S019) of SGI1 was amplified with primers S019Ndefor and sgi_orf019rev, cloned into the SmaI site of pBluescript SK and sequenced (pJKI920), then orf S019 was transferred from pJKI920 into the NdeI-BamHI site of pJKI391, resulting in the expression vector pJKI928. Finally, the Cm^R gene cassette of pAW302 (Stalder and Arber, 1989) was inserted into the SmaI site of the Km^R gene in pJKI928 as a HincII fragment.

pJKI948 is a pJKI780 derivative, where the Cm^R gene cassette of pAW302 (Stalder and Arber, 1989) was inserted as a HincII fragment into the ApaLI site of the Sm^R gene after filling in with Klenow polymerase.

pJKI1023 template plasmid was constructed by replacing the Cm^R gene of pSG76-CS (Kolisnychenko et al., 2002) with the Sm^R/Sp^R gene amplified from pHP45Ω (Prentki and Krisch, 1984). The Sm^R/Sp^R gene was amplified with primers SmRforSmP - SmRrevSmP and cloned into pJKI332 as a PstI fragment replacing the Km^R cassette. After elimination of the XbaI sites of the MCS, the Sm^R/Sp^R gene was transferred from the resulting plasmid, pJKI733, as a NotI-HincII fragment into the NotI-SmaI-digested pSG76-CS.

pMNI41 was generated by digestion of pJKI871 with HindIII followed by religation of the plasmid backbone.

pMSZ934 I-SceI expressing plasmid was created by cloning the 150 bp *oriT* region of plasmid RK2 (positions 51115-51276 bp) from pJKI664 (Kiss et al., 2015) into the SalI site of pSTKST (Kolisnychenko et al., 2002), than the Ap^R cassette amplified from pEMBL19 with primers ampforXSP and amprevXP was cloned into the PstI site of the resulting plasmid pFOL1511.

pMSZ947: The 17710-18681 bp region of SGI1 was amplified from pJKI669 with primers S020promfor_Nc and S022promrev_P. The amplicon was digested with NcoI and PstI and ligated to the appropriate site of pJKI990 (Kiss et al., 2015).

pMSZ948: The 17710-18050 bp region of SGI1 was amplified from pJKI669 with primers S020promfor_Nc and S020promrev. The amplicon was digested with NcoI and EcoRI and ligated to the appropriate site of pJKI990 (Kiss et al., 2015).

pMSZ949: EcoRI-PstI fragment of pJKI780 containing the 16447-18680 bp segment of SGI1 (mob_{SGI1} region) was cloned in the appropriate site of pJKI88.

pMSZ957: The 17801-18680 bp region of SGI1 was amplified from pJKI780 with primers S021for_Stu,Sph and S022promrev. The amplicon was digested with SphI and EcoRI and ligated into the SphI-EcoRI-digested pMSZ949.

pMSZ976 is a deletion derivative of pMSZ949. The protruding ends of EcoRI-SphI digested pMSZ949 were converted to blunt ends using NEBNext® End Repair Module, then religated resulting in pMSZ976 containing the 16447-17805 bp region of SGI1.

pMSZ980: First, the unique TatI site of pJKI88 was eliminated by religation of TatI-digested pJKI88 after blunting the sticky ends using NEBNext® End Repair Module (pMSZ973) then the mob_{SGI1} region was ligated from pJKI783 into the EcoRI-PstI site of pMSZ973, resulting pMSZ978 (analogous to pMSZ949, but without TatI site in the vector). Finally, a deletion was generated by EcoRI-TatI digestion and religation after blunting the sticky ends, which resulted in pMSZ980 carrying the 16447-17881 bp region of SGI1.

pMSZ981: The S020-S019 region was transferred from pJKI876 into the XbaI-PstI site of pJKI88 (pMSZ975) and then the *rrnB* terminator cassette (T1 and T2) was cloned from pJKI988 (Kiss et al., 2015) into the XbaI site of pMSZ975 resulting in pMSZ981.

pMSZ984: The 17713-18140 bp region (S021) of SGI1 was amplified with primers S021for and S021promrev and the EcoRI-PstI-digested amplicon was ligated into the respective site of pJKI88. The resulting plasmid pMSZ974 was digested with EcoRI-BssHII and religated after blunting the sticky ends using NEBNext® End Repair Module leading

to pMSZ977. In this plasmid (containing the 17713-17995 bp region of SGI1), the previous step eliminated the BssHII site, but preserved the EcoRI site. Next, the SphI-PstI fragment (S019-S020) of pJKI783 was ligated into the respective site of pMSZ977 (resulting in pMSZ979). The EcoRI-BssHII fragment of pMSZ979 then was replaced by cloning the EcoRI-BssHII-digested PCR product obtained with primers SGI1orf020_17119for and SGI_17781rev resulting in pMSZ983, which now carried the 16447-17781 bp region of SGI1 (S019-S020+70 bp upstream of S020). Finally, the *rrnB* terminator cassette was cloned from pJKI988 (Kiss et al., 2015) into the BamHI-SalI site of pMSZ983 resulting in pMSZ984.

pMSZ988: The NdeI-PstI fragment of pMSZ981 was replaced by the NdeI-PstI fragment (S019) of pJKI920.

pMSZ989: 18042-18140 bp region (*oriT*ΔIR1) of SGI1 was amplified from pJKI669 with primers oriTd3for and S021promrev. The amplicon was digested with EcoRI-PstI and ligated to the appropriate site of pJK708.

pMSZ990: 18048-18140 bp region (*oriT*ΔIR1) of SGI1 was amplified from pJKI669 with primers oriTd4for and S021promrev. The amplicon was digested with EcoRI-PstI and ligated to the appropriate site of pJK708.

pMSZ991: 18024-18140 bp region (*oriT*) of SGI1 was amplified from pJKI669 with primers oriTd2for and S021promrev. The amplicon containing a spontaneous single base (C) deletion at 18038 bp position in IR1R was digested with EcoRI-PstI and ligated to the appropriate site of pJK708.

pMSZ993: The EcoRI-BssHII-digested PCR product obtained with primers SGI1orf020_17119for and SGI_17732rev was ligated into the EcoRI-BssHII site of pMSZ979 (resulting in pMSZ992), then the *rrnB* terminator cassette was cloned into the BamHI-SalI site of pMSZ992 from pJKI988 (Kiss et al., 2015), resulting in pMSZ993, which contains the 16447-17732 bp region of SGI1 (S019-S020+20 bp upstream of S020).

pMSZ995: The 18024-18140 bp region (*oriT*) of SGI1 was amplified from pJKI669 with primers oriTd2for and S021promrev. The amplicon containing a spontaneous single base (G) deletion at 18037 bp position in IR1R was digested with EcoRI-PstI and ligated to the appropriate site of pJK708.

pMSZ996 The 16447-17712 bp region of SGI1 (S019 and S020 beginning with the second inframe ATG codon) was amplified with primers S020Ndefor and sgi_orf019rev, cloned into the SmaI site of pBluescript SK and sequenced (pJKI876). ORFs S019-S020 then was transferred into pJKI391 as an NdeI-XhoI fragment (pJKI882). The NdeI-BglII fragment of pJKI882 was replaced by cloning the PCR product amplified with primers S020Nde_for2- sgi_S020rev and digested with BglII-NdeI (pMSZ994). Finally, the NdeI-PstI fragment of pMSZ994 was ligated into the NdeI-PstI site of pMSZ981, resulting in pMSZ996, which contains 16447-17619 bp region of SGI1 (S019-S020 until the 2nd inframe ATG codon of S020).

pMSZ997: 18024-18140 bp region (*oriT*) of SGI1 was amplified from pJKI669 with primers oriTd2for and S021promrev. The amplicon was digested with EcoRI-PstI and ligated to the appropriate site of pJK708.

pMSZ1017: 16739-17619 bp region of SGI1 (upstream of S019) was amplified with primers S019promfor and S020Nde_for2. The amplicon was digested with NcoI and ligated to the appropriate site of pJKI990.

$R55^{\Delta Tn\mathit{6187}}$ was generated according to the scarless deletion method (Kolisnychenko et al., 2002). For amplification of the KO PCR fragment, pJKI1023, the Sm^R/Sp^R, Cm^S derivative of pSG76-CS was used as template with primers R55-dTn6187ABfor – R55-dTn6187Crev. The recombination was facilitated by the λ Red recombinase produced from plasmid pJKI842 and the scarless deletion of the Sm^R/Sp^R cassette was promoted by I-SceI, expressed from pMSZ934. The scar site of deletion was then amplified and sequenced using primers R55-dTn6187seqfor and R55-dTn6187seqrev.

Supplementary References

Bolivar, F., Rodriguez, R. L., Greene, P. J., Betlach, M. C., Heyneker, H. L., Boyer, H. W., et al. (1977). Construction and characterization of new cloning vehicles. II. A multipurpose cloning system. *Gene* 2, 95–113. doi:10.1016/0378-1119(77)90000-2.

Datsenko, K. A., and Wanner, B. L. (2000). One-step inactivation of chromosomal genes in Escherichia coli K-12 using PCR products. *Proc. Natl. Acad. Sci. U. S. A.* 97, 6640–5. doi:10.1073/pnas.120163297.

Dente, L., Cesareni, G., and Cortese, R. (1983). pEMBL: A new family of single stranded plasmids. *Nucleic Acids Res.* 11, 1645–1655. doi:10.1093/nar/11.6.1645.

Herrero, M., De Lorenzo, V., and Timmis, K. N. (1990). Transposon vectors containing non-antibiotic resistance selection markers for cloning and stable chromosomal insertion of foreign genes in gram-negative bacteria. *J. Bacteriol.* 172, 6557–6567. doi:10.1128/jb.172.11.6557-6567.1990.

Kiss, J., Nagy, B., and Olasz, F. (2012). Stability, entrapment and variant formation of Salmonella genomic island 1. *PLoS One* 7, e32497. doi:10.1371/journal.pone.0032497.

Kiss, J., and Olasz, F. (1999). Formation and transposition of the covalently closed IS 30 circle : the relation between tandem dimers and monomeric circles. *Mol. Microbiol.* 34, 37–52.

Kiss, J., Papp, P. P. P. P., Szabó, M., Farkas, T., Murányi, G., Szakállas, E., et al. (2015). The master regulator of IncA/C plasmids is recognized by the Salmonella Genomic island SGI1 as a signal for excision and conjugal transfer. *Nucleic Acids Res.* 43, 8735–8745. doi:10.1093/nar/gkv758.

Kolisnychenko, V., Plunkett, G., Herring, C. D., Fehér, T., Pósfai, J., Blattner, F. R., et al. (2002). Engineering a reduced

Escherichia coli genome. *Genome Res.* 12, 640–7. doi:10.1101/gr.217202.
Prentki, P., and Krisch, H. M. (1984). In vitro insertional mutagenesis with a selectable DNA fragment. *Gene* 29, 303–313. doi:10.1016/0378-1119(84)90059-3.
Short, J. M., Fernandez, J. M., Sorge, J. A., and Huse, W. D. (1988). Lambda ZAP: A bacteriophage lambda expression vector with in vivo excision properties. *Nucleic Acids Res.* 16, 7583–7600. doi:10.1093/nar/16.15.7583.
Stalder, R., and Arber, W. (1989). Characterization of in vitro constructed IS30-flanked transposons. *Gene* 76, 187–193. doi:10.1016/0378-1119(89)90159-5.
Vieira, J., and Messing, J. (1982). The pUC plasmids, an M13mp7-derived system for insertion mutagenesis and sequencing with synthetic universal primers. *Gene* 19, 259–268. doi:10.1016/0378-1119(82)90015-4.
