# Supplementary material for: Identification and Characterization of oriT and Two Mobilization Genes Required for Conjugative Transfer of Salmonella Genomic Island 1
Source: Front Microbiol. 2019 Mar 6;10:457. doi: 10.3389/fmicb.2019.00457 (PMC6414798; doi:10.3389/fmicb.2019.00457)
Supplement: Supplementary file 3 [file Data_Sheet_3.PDF]

Table S1. Bacterial strains used in this study.

Strains	Genotype and relevant features[a]	References
E. coli		
TG1	*supE hsd*Δ5 *thi* Δ(*lac-proAB*) F'[*traD36 proAB*+ *lacIq lacZ*ΔM15]	(Gibson, 1984)
TG1Nal	Nal^R derivative of TG1	(Kiss et al., 2012)
TG2	*supE hsd*Δ5 *thi* Δ(*lac-proAB*)Δ(*srl-recA*)306::Tn*10*(Tc^R) F'[*traD36 proAB*+ *lacIq lacZ*ΔM15]	(Sambrook et al., 1989)
TG90	*pcn B80 zad*::Tn*10* (Tc^R) derivative of TG1	(Gonzy-Treboul et al., 1992)
TG90Nal	Nal^R derivative of the TG90, Tc^R, Nal^R	(Kiss et al., 2012)
TG1/R55	TG1 strain containing R55, Ap^R, Cm^R, Flo^R, Sul^R, Km^R, Gm^R	(Kiss et al., 2015)
TG1Nal/R55	TG1Nal strain containing R55, Nal^R, Ap^R, Cm^R, Flo^R, Su^R, Km^R, Gm^R	(Kiss et al., 2015)
TG1Nal/R16a	TG1Nal strain containing R16a, Nal^R, Ap^R, Km^R, Sul^R	(Szabó et al., 2016)
TG1Nal::SGI1-C	TG1Nal strain containing SGI1-C variant integrated into *E. coli thdF*, Nal^R, Sm^R, Sp^R, Sul^R	(Kiss et al., 2015)
TG1Nal::SGI1-C$^{\Delta oriT}$	TG1Nal strain containing the Δ*oriT* mutant SGI1-C, in which the 18016-18140 bp region was replaced, Nal^R, Sm^R, Sp^R, Sul^R	this work
TG1Nal::SGI1-C$^{\Delta S019}$	TG1Nal strain containing the *mpsB* (S019) KO mutant SGI1-C, in which the 16656-16739 bp region was replaced, Nal^R, Sm^R, Sp^R, Sul^R	this work
TG1Nal::SGI1-C$^{\Delta S020}$	TG1Nal strain containing the *mpsA* (*S020*) KO mutant SGI1-C, in which the 17571-17709 bp region was replaced, Nal^R, Sm^R, Sp^R, Sul^R	this work
TG1Nal::mob$_{SGI1}$/R55	TG1Nal::[miniTn*10*::mob$_{SGI1}$-Km^R] mobilization helper strain containing R55 and the 16447-18680 bp SGI1 region integrated into the chromosome by miniTn*10* transposition, Nal^R, Km^R, Ap^R, Cm^R, Flo^R, Sul^R, Gm^R	this work
S17-1 λpir	S17-1 λ*pir*, a λ lysogen derivative of S17-1 (*pro thi recA hsdR* ($r^- m^+$) Tp^R Sm^R Km^S [Ω RP4-2-Tc::Mu-Km::Tn*7*]) expressing Π protein from *pir* gene of R6K	(Simon et al., 1983)
BM14	J5-3 derivative, *pro met azi*; Az^R	Inst.Pasteur, France
Salmonella enterica		
S. Agona 47SA97	SGI1-C^{WT}; $Sm^RSp^RSul^R$	(Boyd et al., 2002)
S. Agona 47SA97 SGI1$^{\Delta int}$	Derivative of *S.* Agona 47SA97 harbouring Δ*int* mutant SGI1-C, $Sm^RSp^RSul^R$	(Doublet et al., 2005)

Table S2. Relevant features of plasmids used in this study.

Plasmid name	Relevant featuresa	References
R55	IncC Type2, tra+, Cm^R, Flo^R, Sul^R, Ap^R, Km^R, Gm^R	(Chabbert et al., 1972)
R55$^{\Delta Tn6187}$	R55 derivative, Tn*6187* was deleted, tra+, Cm^R, Flo^R, Sul^R, Ap^S, Km^S, Gm^S	this work
R16a	IncC Type1, tra+, Ap^R, Km^R, Sul^R	(Chabbert et al., 1972)
R16a$^{\Delta traI}$	IncC Type1, tra-, Δ*traI*::Cm^R, Ap^R, Km^R, Sul^R	(Hegyi et al., 2017)
pACYC184	p15A-based Tc^RCm^R cloning vector	(Rose, 1988)
pBluescript II-SK	pMB1-based Ap^R cloning vector	(Short et al., 198[illegible]
pEMBL19	pMB1-based Ap^R cloning vector	(Dente et al., 198[illegible]
pKD3	R6Kγ-based PCR template plasmid with FRT-flanked *cat* gene for one-step recombination gene-KO Cm^R, Ap^R	(Datsenko and Wanner, 2000)
pKD46	Ap^R ara-inducible expression vector of λ Red recombinase, temperature-sensitive pSC101 replication system	(Datsenko and Wanner, 2000)
pCP20	Thermo-inducible FLP recombinase expression (λ p_R::*FLP*), temperature-sensitive pSC101 replication system, λ *cI857*; Ap^R, Cm^R	(Cherepanov an[illegible] Wackernagel, 1995)
pACYC-1	145-1351 bp region of SGI1 cloned in pACYC184	this work
pACYC-2	1331-2324 bp region of SGI1 cloned in pACYC184	this work
pACYC-3	2354-3874 bp region of SGI1 cloned in pACYC184	this work
pACYC-4	3921-6536 bp region of SGI1 cloned in pACYC184	this work
pACYC-5	6516-8851 bp region of SGI1 cloned in pACYC184	this work
pACYC-6	8860-11754 bp region of SGI1 cloned in pACYC184	this work
pACYC-7	11734-13434 bp region of SGI1 cloned in pACYC184	this work
pACYC-8	13414-15164 bp region of SGI1 cloned in pACYC184	this work
pACYC-9	15144-16913 bp region of SGI1 cloned in pACYC184	this work
pACYC-10	17005-19833 bp region of SGI1 cloned in pACYC184	this work
pACYC-11	19852-21930 bp region of SGI1 cloned in pACYC184	this work
pACYC-12	21981-23590 bp region of SGI1 cloned in pACYC184	this work
pACYC-13	23570-25250 bp region of SGI1 cloned in pACYC184	this work
pACYC-14	25230-28141 bp region of SGI1 cloned in pACYC184	this work
pACYC-10A	17005-18348 bp region of SGI1 cloned in pACYC184	this work

pACYC-10D	17534-18348 bp region of SGI1 cloned in pACYC184	this work
pACYC-10D2	17534-17939 bp region of SGI1 cloned in pACYC184	this work
pACYC-10D4	17799-18348 bp region of SGI1 cloned in pACYC184	this work
pACYC-10D4B	18017-18348 bp region of SGI1 cloned in pACYC184	this work
pACYC-10D4C	18132-18348 bp region of SGI1 cloned in pACYC184	this work
pACYC-10D4E	18017-18261 bp region of SGI1 cloned in pACYC184	this work
pACYC-10D7	18017-18151 bp region of SGI1 cloned in pACYC184	this work
pFOL1343	Sm^R derivative of pJKI671.	this work
pFOL1362	15444-22496 bp (S015-S025) region of SGI1 cloned in pJK708.	this work
pFOL1365	BglII deletion derivative of pFOL1362 containing 15444-16807 bp and 21056-22496 bp regions of SGI1.	this work
pFOL1372	EcoRI deletion derivative of pFOL1362 containing 15444-19843bp (S015-S023) region of SGI1.	this work
pJKI88	p15A-based Km^R cloning vector deriving from pACYC177 (Rose, 1988).	(Kiss and Olasz, 1999)
pJKI391	p15A-based Km^R expression vector deriving from pJKI88.	(Kiss et al., 2015
pJKI669	d1 deletion derivative of SGI1-C (SGI1-C-d1, (Kiss et al., 2012)) containing DRL-S004 and S013-DRR regions of SGI1-C cloned in the pJKI88-derived vector pJKI633.	this work
pJKI672	BssHII deletion derivative of pJKI669 containing DRL-S004, S019 and *intI1*-DRR regions of SGI1-C.	this work
pJKI678	MfeI deletion derivative of pJKI669 containing DRL and S025-DRR regions of SGI1-C.	this work
pJKI708	Sm^R derivative of the p15A-based cloning vector, pJKI88	(Hegyi et al., 2017)
pJKI710	16807-19427 bp (S020-S023) region of SGI1 cloned in pJK708.	this work
pJKI725	PstI-SacI deletion derivative of pFOL1372 containing the 16594-19843 bp (S020-S023) region of SGI1.	this work
pJKI726	15439-16595 bp (S015-S019) region of SGI1 cloned in pJK708.	this work
pJKI731	15439-18050 bp (S015-S021) region of SGI1 cloned in pJK708.	this work
pJKI737	pFOL1372 derivative carrying KO mutation in S020 in the15444-19843bp (S015-S023) region of SGI1.	this work
pJKI772	pFOL1372 derivative carrying KO mutation in S019 in the15444-19843bp (S015-S023) region of SGI1.	this work
pJKI774	pFOL1372 derivative carrying KO mutation in S022 in the15444-19843bp (S015-S023) region of SGI1.	this work
pJKI775	15439-18680 bp (S015-S022) region of SGI1 cloned in pJK708.	this work
pJKI776	15849-18680 bp (S018-S022) region of SGI1 cloned in pJK708.	this work
pJKI777	16087-18680 bp (S019-S022) region of SGI1 cloned in pJK708.	this work
pJKI780	16447-18680 bp mob$_{SGI1}$ region (S019-S022) of SGI1 cloned in pJK708.	this work
pJKI781	16447-18140 bp (S019-S021) region of SGI1 cloned in pJK708.	this work
pJKI791	18304-18680 bp of SGI1 (upstream region of S022) cloned in pJK708.	this work
pJKI796	pLOFKm (Herrero et al., 1990) derivative R6K-based delivery plasmid containing the 16447-18680 bp (S019-S022) region of SGI1 with a Km^R gene in the transposable mini-Tn*10* unit. R6Kγori, *oriT*$_{RK2}$, Ap^R, *lacI*^{q}, P$_{tac}$::Tn*10* transposase, miniTn*10*::SGI1$_{16447-18680}$-Km^R.	this work
pJKI810	17713-18140 bp region of SGI1 cloned in pJK708.	this work
pJKI811	17713-18050 bp region of SGI1 cloned in pJK708.	this work
pJKI818	17961-18140 bp region of SGI1 cloned in pJK708.	this work
pJKI833	Cm^R, Sm/Sp^S derivative of pJKI737 carrying KO mutation in *mpsA* in the15444-19843bp (S015-S023) region of SGI1.	this work
pJKI835	Cm^R, Sm/Sp^S derivative of pJKI772 carrying KO mutation in *mpsB* in the15444-19843bp (S015-S023) region of SGI1.	this work
pJKI836	Cm^R, Sm/Sp^S derivative of pFOL1372.	this work
pJKI842	Tc^R, Ap^S derivative of pKD46, the ara-inducible expression vector of λ Red recombinase with temperature-sensitive pSC101 replication system	this work
pJKI871	18016-18140 bp region of SGI1 (*oriT*$_{SGI1}$) cloned in pJK708.	this work
pJKI872	18016-18119 bp region of SGI1 (*oriT*ΔIR3R) cloned in pJK708. The truncated *oriT* lacks the right copy of IR3.	this work
pJKI873	18016-18140 bp region of SGI1 (*oriT*ΔIR2R) cloned in pJK708. The right copy of IR2 in *oriT* is eliminated by base substitutions.	this work
pJKI874	18035-18140 bp region of SGI1 (*oriT*ΔIR1L) cloned in pJK708. The truncated *oriT* lacks the left copy of IR1.	this work
pJKI935	Cm^R pJKI391 derivative p15A-based vector expressing *mpsA* under the control of P$_{tac}$ promoter	this work
pJKI937	Cm^R pJKI391 derivative p15A-based vector expressing *mpsB* under the control of P$_{tac}$ promoter	this work
pJKI948	16447-18680 bp mob$_{SGI1}$ region (S019-S022) of SGI1, a Cm^R derivative of pJKI780	this work
pJKI990	ColE1-based cloning vector for β-gal assays, containing promoterless *lacZ* gene preceded by pHP45Ω (Prentki and Krisch, 1984) and *rrnB* terminators.	(Kiss et al., 2015
pJKI1023	Sm^R/Sp^R derivative of the R6Kγ-based PCR template plasmid pSG76-CS (Kolisnychenko et al., 2002), where I-SceI cleavage sites flank the resistance cassette.	this work
pMNI41	Km^R derivative of pJKI871, carrying the *oriT*$_{SGI1.}$	this work
pMSZ934	Ap^R, Km^R mobilizable derivative of the I-SceI producer plasmid pSTKST (Kolisnychenko et al., 2002). Temperature-sensitive pSC101 replication system, Tc^R, P$_{tet}$::SCEI, *oriT*$_{RK2}$	this work
pMSZ947	pJKI990-derivative β-galactosidase tester plasmid containing the non-coding upstream region of *mpsA* (17710-18681 bp) fused to the promoterless *lacZ* gene.	this work
pMSZ948	pJKI990-derivative β-galactosidase tester plasmid containing the non-coding upstream region of *mpsA* (17710-18050 bp) fused to the promoterless *lacZ* gene.	this work
pMSZ949	16447-18680 bp mob$_{SGI1}$ region (S019-S022) of SGI1 cloned in pJKI88, a Km^R equivalent of pJKI780.	this work
pMSZ957	16447-18680 bp mob$_{SGI1}$ region (S019-S022) of SGI1 cloned in pJKI88, a Km^R equivalent of pJKI780. pMSZ957 contains a single T insertion at 17816th position, which generates a new StuI site and cause frameshift in S021.	this work
pMSZ976	16447-17805 bp region of SGI1 (*mpsAB*+94 bp upstream of *mpsA*) cloned in pJKI88.	this work
pMSZ980	16447-17881 bp region of SGI1 (*mpsAB*+170 bp upstream of *mpsA*) cloned in the pJKI88-analogue pasmid pMSZ973.	this work
pMSZ981	16447-17712 bp region of SGI1 (*mpsAB*) cloned in pJKI88.	this work
pMSZ984	16447-17781 bp region of SGI1 (*mpsAB*+70 bp upstream of *mpsA*) cloned in pJKI88.	this work
pMSZ988	16447-16743 bp region of SGI1 (*mpsB*) cloned in pJKI88.	this work
pMSZ989	18042-18140 bp region (*oriT* ΔIR1) of SGI1 cloned in pJK708. The truncated *oriT*$_{SGI1}$ fragment lacks the IR1 repeat.	this work
pMSZ990	18048-18140 bp region (*oriT* ΔIR1+spacer to IR2L) of SGI1 cloned in pJK708. The truncated *oriT*$_{SGI1}$ fragment lacks the IR1 repeat and the 6-bp spacer sequence to IR2L.	this work
pMSZ991	18024-18140 bp region *oriT*$_{SGI1}$ cloned in pJK708. This fragment lacks the 7 bp preceding IR1L and contains a single base (C) deletion at 18038 bp position in IR1R.	this work
pMSZ993	16447-17732 bp region of SGI1 (*mpsAB*+20 bp upstream of *mpsA*) cloned in pJKI88.	this work
pMSZ995	18024-18140 bp region *oriT*$_{SGI1}$ cloned in pJK708. This fragment lacks the 7 bp preceding IR1L and contains a single base	this work

	(G) deletion at 18037 bp position in IR1R.	
pMSZ996	16447-17619 bp region of SGI1 (*mpsB-mpsA* beginnig with the 2nd inframe ATG codon) cloned in pJKI88.	this work
pMSZ997	18024-18140 bp region $oriT_{SGI1}$ cloned in pJK708. This fragment lacks the 7 bp preceding IR1L.	this work
pMSZ1017	pJKI990-derivative β-galactosidase tester plasmid containing the upstream region of *mpsB* (16741-16975 bp) fused to the promoterless *lacZ* gene.	this work

Table S3. List of oligonucleotides used.

**Primer**	**Sequence (5'-3')a**	**Reference**
ampforXSP	gctctagagtcgacctgcagtacattcaaatatgtatccgctc	this work
amprevXP	aatctagactgcagggtctgacagttaccaatgc	this work
attsgi1for	gctctagagcggccgcatggaaggcggcttcctggc	(Kiss et al., 2012)
attsgi1rev	gctctagagcggccgcaaatgtgaatcgaatcacaatcg	(Kiss et al., 2012)
deloriTfor	gtgtcattctttggaaaggaaagcgcgaagcgcgctaaccgccgaaggcgGTGTAGGCTGGAGCTGCTTC	this work
deloriTrev	aagagggcctccctcatccgtcagaacgagtgctggatttccggcttactCATATGAATATCCTCCTTAGTTC	this work
delS019for	gtgatggtgcgttgactccttttttcattggcggcaatatcgtttagagccGTGTAGGCTGGAGCTGCTTC	this work
delS019rev	gggtcatggtcgcatcgatgtgacaaatagttatttggggtaattgatggCATATGAATATCCTCCTTAGTTC	this work
delS020for	tatgtcccgcatgtcctttatgccctgttcggacttaatccaagcgctaaGTGTAGGCTGGAGCTGCTTC	this work
delS020rev	acgggccaacaaagtaacaattttgattaacagagttagggggatcaatgCATATGAATATCCTCCTTAGTTC	this work
delS022for	gtgtttggccgctcggaattgagcgccaggaacgcgacatagctgtcagcGTGTAGGCTGGAGCTGCTTC	this work
delS022rev	aggggcactcctccgtctaaaacctatctccccggaggaaaatcagatgtCATATGAATATCCTCCTTAGTTC	this work
FwEcoRI1	attgtgaattcttctgtattgggaagtaaat	this work
FwEcoRI10	attgtgaattcgtaggcttcttgccgaaact	this work
FwEcoRI10D	attgtgaattcctttatgccctgttcggact	this work
FwEcoRI10D3A	attgtgaattcctctgcatgctaaggccaac	this work
FwEcoRI10D4B	tattgtgaattctataattcgcgcacattcgt	this work
FwEcoRI10D4C	tattgtgaattcggagcatagagtaagccgga	this work
FwEcoRI11	attgtgaattcaagaaaacatcgctgtaagt	this work
FwEcoRI12	attgtgaattcacgcttgagttatcttcttc	this work
FwEcoRI13	attgtgaattcatatctaagttgttaccggc	this work
FwEcoRI14	attgtgaattcctctgatgcatcttgctcta	this work
FwEcoRI2	attgtgaattcaaaacatgttactttccacg	this work
FwEcoRI3	attgtgaattcgccagtcactttcttaactt	this work
FwEcoRI4	attgtgaattcaccaaagttaaggccaagtt	this work
FwEcoRI5	attgtgaattcaacacatgttcgctgattaa	this work
FwEcoRI6	attgtgaattcgtcaccatattgctgaacaa	this work
FwEcoRI7	attgtgaattcgttgtacgcgtcctcaatag	this work
FwEcoRI8	attgtgaattctctcatactgttgctccaat	this work
FwEcoRI9	attgtgaattctagaccagatcttggagcat	this work
oriTd1for	aactgcagtgcgcggtgcgaaagcc	this work
oriTd2for	aactgcagcgcgcacattcgtgcgcggtgcgaaag	this work
oriTd2rev	ttgaattcgccgtaggcgtaaacagag	this work
oriTd3for	aactgcagtgcgaaagcctagagcccttg	this work
oriTd4for	aactgcagaagcctagagcccttgaggc	this work
oriTfor	aactgcagtataattcgcgcacattcgtg	this work
oriTIR2mutrev	cgacggaagcttcactcctcaagggc	this work
pBRTcPstfor	aactgcaggcgtatcacgaggccctttc	this work
pBRTcPstrev	aactgcagtggtgaatccgttagcgagg	this work
R55_dTn6187seqfor	aactgcaggcgttttgcagcgttc	this work
R55-dTn6187ABfor	gtcttcgcagttcgccagctttccaacgcatggaaagttaccccttctgatgccatcgcgctgctacgccaatcagcgtggatggaccgaaatttgtccTCAACAGGTT GAACTGCGGATC	this work
R55-dTn6187Crev	ctcagaaaacggaatctatggtcactcccgtttttgcaacaccgattttgGATTTAGGTGACACTATAGAATAC	this work
R55-dTn6187seqrev	tagtcgacagatttagaccatcatgcaacg	this work
RvNcoI1	attgtccatggcgtggaaagtaacatgtttt	this work
RvNcoI10	attgtccatggacggatcagcaatatgaact	this work
RvNcoI10A	attgtccatggatctccccggaggaaaatca	this work
RvNcoI10D2	attgtccatggtttgttccgtcggaacagac	this work
RvNcoI10D3	attgtccatggtccggcttactctatgctcc	this work
RvNcoI10D4D	attgtccatggaggcttccatatcgcgatct	this work
RvNcoI11	attgtccatggggtacggtatcgcctaagtg	this work
RvNcoI12	attgtccatgggccggtaacaacttagatat	this work
RvNcoI13	attgtccatggtagagcaagatgcatcagag	this work
RvNcoI14	attgtccatggaatatcgctgtatggcttca	this work
RvNcoI2	attgtccatggattccataccgtaattgact	this work
RvNcoI3	attgtccatggggatattgattggagtaagg	this work
RvNcoI4	attgtccatggttaatcagcgaacatgtgtt	this work
RvNcoI5	attgtccatggcagatagcaatggatactgc	this work
RvNcoI6	attgtccatggctattgaggacgcgtacaac	this work
RvNcoI7	attgtccatggattggagcaacagtatgaga	this work
RvNcoI8	attgtccatggatgctccaagatctggtcta	this work
RvNcoI9	attgtccatggcggtaaaagcaggtgttaaa	this work
S019Ndefor	gatgatcatatggaaaaatgggtaagagaaagaagc	this work
S019promfor	atccatggattaccccaaataactatttgtcacatcg	this work
S020for1	tactcatcctgcaggcttttaaac	this work
S020for2	aactgcagtcgatacataaaatctccctc	this work

S020for3	aactgcagaatcgaagccttatttagtag	this work
S020for4	aactgcagttaatcagcagagccggtgtttttg	this work
S020Nde_for2	gatgatcatatgaagagttttcagtccatagcc	this work
S020Ndefor	gatgatcatatgcgttcagagcggactaatccggat	this work
S020promfor_Nc	aaccatggatccccctaactctgttaatc	this work
S020promrev	aagaattctttcgcaccgcgcacgaatg	this work
S021for	aactgcagtgatccccctaactctgttaatc	this work
S021for_Stu,Sph	ctgcatgctaaggcctaacgctgggatc	this work
S021for2	ccggctgcagctgtcgtgtcattctttgg	this work
S021promrev	aagaattcctatgctccaccccgtctctg	this work
S022promfor	cactgcagtgcaagcacaatgatgataaacatc	this work
S022promrev	aagaattcgtaagttagttgagcatccaaac	this work
S022promrev_P	aactgcagtaagttagttgagcatccaaac	this work
sgi_17781rev	gtgaattcggatccgtcgactttcacttaccaccggatacggac	this work
sgi_orf019rev	aactgcagggatccttaatcagcagagccggtgtttttg	this work
sgi_orf020for	aacatatgcgttcagagcggactaatc	this work
sgi_S020rev	aaggatccttaccccaaataactatttgtcac	this work
SGI1orf020_17119for	gaacagtgcgcgcccggccac	this work
SmRforSmP	cgctgcagcccggggttgccgggtgacgcac	this work
SmRrevSmP	aactgcagcccgggtcggcttgaacgaattgttagac	this work

[a] Uppercase shows the template plasmid sequence in primers used fro producing the KO amplicons. Restriction cleavage sites are underlined.

Supplementary References

Boyd, D., Cloeckaert, a., Chaslus-Dancla, E., and Mulvey, M. R. (2002). Characterization of Variant Salmonella Genomic Island 1 Multidrug Resistance Regions from Serovars Typhimurium DT104 and Agona. *Antimicrob. Agents Chemother.* 46, 1714–1722. doi:10.1128/AAC.46.6.1714-1722.2002.

Chabbert, Y. A., Scavizzi, M. R., Witchitz, J. L., Gerbaud, G. R., and Bouanchaud, D. H. (1972). Incompatibility Groups and the Classification of f- Resistance Factors. *J. Bacteriol.* 112, 666–675.

Cherepanov, P. P., and Wackernagel, W. (1995). Gene disruption in Escherichia coli: TcR and KmR cassettes with the option of Flp-catalyzed excision of the antibiotic-resistance determinant. *Gene* 158, 9–14. doi:10.1016/0378-1119(95)00193-A.

Datsenko, K. A., and Wanner, B. L. (2000). One-step inactivation of chromosomal genes in Escherichia coli K-12 using PCR products. *Proc. Natl. Acad. Sci. U. S. A.* 97, 6640–5. doi:10.1073/pnas.120163297.

Dente, L., Cesareni, G., and Cortese, R. (1983). pEMBL: A new family of single stranded plasmids. *Nucleic Acids Res.* 11, 1645–1655. doi:10.1093/nar/11.6.1645.

Doublet, B., Boyd, D., Mulvey, M. R., and Cloeckaert, A. (2005). The Salmonella genomic island 1 is an integrative mobilizable element. *Mol. Microbiol.* 55, 1911–1924. doi:10.1111/j.1365-2958.2005.04520.x.

Gibson, T. J. (1984). Studies on the Epstein-Barr virus genome. Thesis.

Gonzy-Treboul, G., Karmazyn-Campelli, C., and Stragier, P. (1992). Developmental regulation of transcription of the Bacillus subtilis ftsAZ operon. *J. Mol. Biol.* 224, 967–979. doi:10.1016/0022-2836(92)90463-T.

Hegyi, A., Szabó, M., Olasz, F., and Kiss, J. (2017). Identification of oriT and a recombination hot spot in the IncA/C plasmid backbone. *Sci. Rep.* 7, 10595. doi:10.1038/s41598-017-11097-0.

Herrero, M., De Lorenzo, V., and Timmis, K. N. (1990). Transposon vectors containing non-antibiotic resistance selection markers for cloning and stable chromosomal insertion of foreign genes in gram-negative bacteria. *J. Bacteriol.* 172, 6557–6567. doi:10.1128/jb.172.11.6557-6567.1990.

Kiss, J., Nagy, B., and Olasz, F. (2012). Stability, entrapment and variant formation of Salmonella genomic island 1. *PLoS One* 7, e32497. doi:10.1371/journal.pone.0032497.

Kiss, J., and Olasz, F. (1999). Formation and transposition of the covalently closed IS 30 circle : the relation between tandem dimers and monomeric circles. *Mol. Microbiol.* 34, 37–52.

Kiss, J., Papp, P. P. P. P., Szabó, M., Farkas, T., Murányi, G., Szakállas, E., et al. (2015). The master regulator of IncA/C plasmids is recognized by the Salmonella Genomic island SGI1 as a signal for excision and conjugal transfer. *Nucleic Acids Res.* 43, 8735–8745. doi:10.1093/nar/gkv758.

Kolisnychenko, V., Plunkett, G., Herring, C. D., Fehér, T., Pósfai, J., Blattner, F. R., et al. (2002). Engineering a reduced Escherichia coli genome. *Genome Res.* 12, 640–7. doi:10.1101/gr.217202.

Prentki, P., and Krisch, H. M. (1984). In vitro insertional mutagenesis with a selectable DNA fragment. *Gene* 29, 303–313. doi:10.1016/0378-1119(84)90059-3.

Rose, R. E. (1988). The nucleotide sequence of pACYC184. *Nucleic Acids Res.* 16, 355. doi:10.1093/nar/16.1.356.

Sambrook, J., Fritsch, E. F., and Maniatis, T. (1989). *Molecular Cloning: A Laboratory Manual. Cold Spring Harbor Laboratory Press, Cold Spring Harbor, NY.*

Short, J. M., Fernandez, J. M., Sorge, J. A., and Huse, W. D. (1988). Lambda ZAP: A bacteriophage lambda expression vector with in vivo excision properties. *Nucleic Acids Res.* 16, 7583–7600. doi:10.1093/nar/16.15.7583.

Simon, R., Priefer, U., and Pühler, A. (1983). A Broad Host Range Mobilization System for In Vivo Genetic Engineering: Transposon Mutagenesis in Gram Negative Bacteria. *Bio/Technology* 1, 784–791. doi:10.1038/nbt1183-784.

Szabó, M., Nagy, T., Wilk, T., Farkas, T., Hegyi, A., Olasz, F., et al. (2016). Characterization of Two Multidrug-Resistant IncA/C Plasmids from the 1960s by Using the MinION Sequencer Device. *Antimicrob. Agents Chemother.* 60, 6780–6786. doi:10.1128/AAC.01121-16.
